# Enhancing physical performance with ischemic preconditioning: a systematic review and meta-analysis of moderators and performance outcomes

**DOI:** 10.5114/biolsport.2026.154945

**Published:** 2025-10-31

**Authors:** Yilin Zhang, Kai Xu, Hao Kong, Mingyue Yin, Chenghao Liu, Yun Xie, Liam Kilduff, Gustavo R Mota, Olivier Girard

**Affiliations:** 1 School of Sports Training, Tianjin University of Sport, No.16 Donghai Road, Tuanbo New Town West, Jinghai District, Tianjin, 301617, China; 2 School of Medical and Healt Sciences, Centre for Human Performance, Edith Cowan University, Joondalup, Australia; 3 The Mar MacKillop Institute for Health Research, Australian Catholic University, Melbourne,VIC. 3000, Australia; 4 A-STEM, School of Engineering, Swansea University, Swansea, UK; 5 Welsh Institute of Performance Science (WIPS), Swansea University Performance Science, Swansea, UK; 6 Exercise Science, Health and Human Performance Research Group, Department of Sport Sciences, Institute of Health Sciences, Federal University of Triângulo Mineiro, Uberaba/MG, Brazil; 7 School of Human Science (Exercise and Sport Sciences), The University of Western Australia, Perth, Western Australia, Australia

**Keywords:** Blood flow restriction, Exercise performance, Ergogenic aids, Warm-up protocols, Time window, Training level, Placebo

## Abstract

This study examined the effects of ischemic preconditioning (IPC) on physical performance, considering the influence of timing, warm-up (WU), participant characteristics, and IPC protocols structure. A total of 90 trials (1,439 participants) were retrieved from three databases and assessed using PICOS criteria. Multilevel meta-analyses with cluster-robust variance adjustments were conducted to calculate pooled effect sizes (Hedge’s g). Risk of bias and certainty of evidence were assessed using the RoB 2 tool and GRADE framework. IPC produced a trivial but significant improvement in physical performance (g = 0.13, P < 0.01), which persisted after excluding SHAM effects (g = 0.10; P < 0.01). Significant improvements were observed for maximum repetitions, time to failure, and power output, but not for jump ability, strength, or oxygen uptake. Comparable benefits were found for anaerobic (g = 0.15) and aerobic (g = 0.10) exercise, with greater effects in males and less-trained participants. Performance was further enhanced when IPC was followed by WU (g = 0.16), with the optimal IPC-to-WU interval being ~42 minutes. Without WU, the effective IPC window narrowed to 6–7 minutes. In practice, IPC can enhance physical performance, independently of sham effects, moderated by sex, training level, and WU. For competition or testing, the most effective strategy appears to be 3 or 4 × 5-min IPC protocol, followed by a 42-min interval, standardized warm-up, and endurance testing. For mechanistic studies, WU should be excluded, and testing conducted 6-7 minutes post-IPC. Future research should target women, middle-aged individuals, and elite athletes.

## INTRODUCTION

Researchers have long sought optimal interventions to enhance physical performance [1–4]. Ischemic preconditioning (IPC) has emerged as a promising intervention, showing significant physiological effects, such as reducing ischemia-induced ultrastructural damage and delaying irreversible myocardial injury [5, 6]. Additionally, IPC has been shown to decrease energy metabolism rates [6], enhance tolerance to subsequent ischemia [7], and preserve adenosine triphosphate (ATP) levels through intermittent reperfusion [8]. Collectively, these mechanisms support the potential of IPC as a viable non-pharmacological intervention for enhancing athletic performance.

Despite decades of research, the efficacy of IPC in enhancing physical performance remains controversial [9]. Some studies report improvements in anaerobic performance, such as short-duration swimming [10, 11], the maximal number of repetitions (RM) in high-load resistance exercises [12, 13], and performance in exhaustion tests [14, 15]. IPC has also been shown to enhance aerobic performance in tasks such as 5-km runs [16, 17], cycling time trials [18], incremental tests [18–20], and supramaximal intensity tests [21], while also increasing maximal oxygen uptake (${\dot{\text{V}}\text{O}}_{2\max}$) [20, 21]. However, other research reports no significant performance enhancement following IPC in anaerobic-type efforts such as sprinting [22, 23], time trials [24, 25], jump ability [26], maximal voluntary contraction (MVC) force [27, 28], or one-repetition maximum (1RM) [29]. Likewise, IPC has not consistently improved aerobic-like efforts including time trials [30–34], time to exhaustion [35, 36], or ${\dot{\text{V}}\text{O}}_{2\max}$ [30, 37, 38].

Research suggest that IPC may exert a greater influence on aerobic than anaerobic physical performance. In their systematic review, Caru et al. [2] concluded that IPC enhances aerobic performance, primarily through improved oxygen utilization and reduced blood lactate production. A recent meta-analysis also demonstrated that IPC significantly reduces task completion time in activities predominantly reliant on aerobic metabolism and tends to extend time to exhaustion [39]. Although no significant improvement in ${\dot{\text{V}}\text{O}}_{2\max}$ was observed, this may be attributed to the already high training levels of participants, whose ${\dot{\text{V}}\text{O}}_{2\max}$ values were likely approaching their genetic ceiling [39]. Highly trained athletes may exhibit a diminished response to IPC, while individuals with lower training levels may derive greater benefits. However, these assumptions require confirmation through larger, well-controlled studies.

The time interval between IPC application and subsequent exercise testing is crucial to its efficacy [2]. That said, reported time intervals in the literature vary widely – from immediate to several hours – with no clear consensus. This inconsistency may stem from inconsistent reporting and a failure to account for the additive effects of WU included within the IPC-to-test timeline. For example, Kaur et al. [38] reported a 15-min interval between IPC and testing, but this actually included a 3-min WU and 2 min of measurement, extending the real interval to 20 min. Lisbôa et al. [40] found no performance improvement in a 50-m sprint swim 1-h post-IPC, yet significant improvements emerged at 2 and 8-h (i.e., intervals that also included a 20-min WU). Similarly, Richard et al. [25] reported a 1-h interval from IPC to testing, which also included a 20-40-min WU. These inconsistencies highlight the need for a systematic evaluation of IPC timing, as no standardized protocol currently exists. Establishing the optimal IPC-to-exercise interval is essential to maximize potential performance-enhancing effects and reduce variability across studies.

Previous meta-analyses have not fully accounted for key moderating factors, such as sex, age, standardized IPC and exercise protocols, and the additive effects of WU [39, 41, 42]. Controlling for WU is critical, as it is a well-established performance enhancer. For example, Souza et al. [41] reported IPC improved performance in exhaustion tests (standardized mean difference [SMD] = 0.51) and RM (SMD = 0.97) compared to CON, but not *versus* sham. This suggests that the observed IPC benefits may partly overlap with the effects of WU, and inadequate control could underestimate of IPC’s true effects beyond sham. Although Chen et al. [39] demonstrated that IPC could improve physical performance, such as enhancing exhaustive test results and reducing task completion time, they did not account for factors such as sham effect or the varying time intervals between IPC and testing, which ranged from immediate to 24 h in the included studies [39, 41]. This indicates that even recent metaanalyses have overlooked the importance to standardize interval times, despite previous research highlighting their variability [2]. Therefore, standardized protocols for both exercise and IPC interventions, particularly regarding WU and timing intervals, are needed to accurately isolate the true effects of IPC [41, 42].

The limitations of existing literature extend beyond IPC interval time definition to include methodological heterogeneity and demographic variables. For example, Souza et al. [41]’s recent meta-analysis extracted only one data point per study to avoid duplicate calculations, which likely underestimated the true effect size (ES), as multi-level meta-analyses can better account for multiple datasets within a single study [43–45]. Additionally, factors such as the inclusion of a SHAM control group, the incorporation of a WU, and ischemia-reperfusion parameters (e.g., duration) can significantly influence IPC outcomes. For instance, the sham effect may overestimate the true efficacy of IPC [41]. Demographic factors such as sex and age may also play a role. For example, Teixeira et al. [46] found sex differences in functional sympathetic responses during grip strength exercises following IPC. Specifically, IPC significantly enhanced functional sympatholysis in males (Δ–3% ± 9% *vs.* baseline Δ–8% ± 7%, *P* = 0.02) but not in females (Δ–5% ± 10% *vs.* baseline Δ–8% ± 9%, *P* = 0.13). Similarly, Pereira et al. [15] showed improved time to exhaustion after IPC in males only. Age-related physiological changes, such as reduced vascular elasticity and metabolic capacity, may also affect the efficacy of IPC in older populations. Despite extensive research, controversies persist regarding the optimal IPC protocols, particularly the timing of intervention, its effects on different exercise types (aerobic *vs.* anaerobic), and how athletes’ training levels influence outcomes.

This study aims to evaluate the effects of IPC on physical performance and optimize its application protocols through meta-analysis and systematic review. The specific objectives include: (1) assessing the physical performance effects of IPC on anaerobic and aerobic performance; and (2) exploring potential moderators including participant characteristics (training level, sex, age) and study design factors (time intervals, sham effects, ischemia-reperfusion parameters, WU protocols, and exercise types) on IPC efficacy through subgroup and regression analyses.

## MATERIALS AND METHODS

### Registration of Systematic Review Protocol

The systematic review followed the Preferred Reporting Items for Systematic Reviews and Meta-Analyses (PRISMA) 2020 guidelines (Supplementary Material) [47]. The study was retrospectively registered in PROSPERO (CRD42024614265) after the initial data analysis and chart preparation, but before the secondary search and re-analysis.

### Search Strategy

Before the formal search, one author (Y.L.Z) conducted an abbreviated search. A systematic literature search was then performed by two independent reviewers (K.X and M.Y.Y) in PubMed (MEDLINE), Web of Science, and EMBASE, ending on 4 September 2024. The search terms included a combination of database specific MeSH terms and keywords: “remote ischemic preconditioning” OR “remote ischaemic preconditioning” OR “remote preconditioning” OR “remote conditioning” OR “remote ischemic conditioning” OR “remote ischaemic conditioning” OR “transient limb ischemia” OR “muscle ischemia” OR “ischemic preconditioning” AND “performance” OR “sport*” OR “exercise” OR “strength training” OR “running” OR “swimming” OR “cycling” OR “athletes” OR “athletic performance”. When possible, human filters were added in each database (Detailed search terms for the different databases are provided in Supplementary Material). Additionally, reference lists of all identified studies were manually scanned for additional studies.

### Study Selection and Eligibility Criteria

One author (Y.L.Z) performed the literature identification and screening. During the eligibility phase, two authors (K.X and M.Y.Y) reviewed the full texts, applying the exclusion and inclusion criteria. In cases of uncertainty, a third author (Y.L.Z) made the final decision. All processes were conducted using Zotero. The eligibility criteria, based on the “PICOS” framework (P, participants; I, intervention; C, control; O, outcome; S, study), are provided in Table 1.

**TABLE 1:** Inclusion and Exclusion criteria.

**Inclusion criteria**
**P**: Include healthy subjects of all ages (from adolescents to older adults) and covering a wide range of physical activity levels, from sedentary individuals to world-class athletes.
**I**: Intervention involves IPC combined with exercise, applied solely before exercise, not during or post-exercise/testing. Crossover studies must include CON, SHAM, and IPC groups. Groups without IPC were considered as CON, those with very low pressure (e.g., 20 mmHg) were considered as SHAM, and IPC was defined as the application of regular occlusion pressure, such as 220 mmHg.
**C**: Control groups for IPC must be SHAM (using lower pressure values) or CON (no ischemic conditions).
**O**: Include outcomes related to physical performance: balance, jump, strength, MAOD (maximal accumulated oxygen deficit), power output,repetitions, time trial performance, time-toexhaustion tests, and ${\dot{\text{V}}\text{O}}_{2}$.
**S**: Include randomized controlled trials and randomized crossover studies.

**Exclusion criteria**
**A.** Exclude studies involving subjects with any medical conditions.
**B.** Exclude studies applying IPC during or after exercise/testing.
**C.** Studies combining IPC with other interventions (e.g., caffeine, hypoxia) are not excluded if they include IPC vs. CON/SHAM comparisons.
**D.** Exclude non-English publications.
**E.** Exclude non-human studies.
**F.** Exclude studies lacking physical performance outcomes.
**G.** Exclude reviews, meta-analyses, and conference abstracts.
**H.** Exclude non-IPC interventions (e.g., blood flow restriction) and long-term IPC interventions.
**I.** Exclude duplicate publications of the same experiment; retain only one version.

IPC, Ischemic preconditioning; SHAM/CON, SHAM /Control Group;

### Risk of Bias Assessment

The Cochrane Risk of Bias 2 (ROB2 IRPG beta and ROB2 crossover beta) tool was used to assess the risk of bias in randomized controlled trials (RCTs) and randomized crossover trial (RC) studies meeting the inclusion criteria [48]. The tool evaluates five domains: (D1) randomization process, (DS) period/carryover effects, (D2) deviation from intended intervention (assignment effects and adherence), (D3) missing outcome data, (D4) outcome measurement, and (D5) selection of the reported result. Each domain was rated as “yes,” “probably yes,” “probably no”, or “no”. Bias risk was classified as “high”, “some concerns”, or “low” according to Cochrane’s guidelines [49]. A study was rated “high risk” if at least one domain was high risk, “some concerns” if at least one domain raised concern but none were high risk, and “low” if all domains were rated low [49]. Two reviewers independently assessed the domains and physical performance bias qualification for each study, with discrepancies resolved by a third reviewer.

### Data extraction and collation

We meticulously extracted study characteristics including participants’ sex, age, exercise experience, the number and duration of IPC cycles, cuff pressure, limb location, WU content and intensity, IPC timing relative to WU and testing, interval times between different IPC timings, exercise types, and pre- and post-test outcome data for IPC, SHAM, and CON groups. We strictly defined interval times, specifying whether they referred to the period from IPC to testing or from IPC to WU. This distinction was necessary as some studies included WU within the interval, while others did not.

Studies were categorized as anaerobic and aerobic based on exercise duration (75 s) [50, 51]. For each outcome, three reviewers carefully reviewed the exercise protocols to determine the total duration. Exercises lasting more than 75 seconds were classified as aerobic, while those lasting 75 seconds or less were classified as anaerobic. Studies including both types were categorized separately. Classifications were conducted by H.K. and K.X., with disagreements resolved by Z.Y.L. The exercise experience level of participants was classified into six categories (0–5) according to McKay et al. [52]’s criteria: sedentary, recreationally active, developing or trained, highly trained or national level, elite or international level, and worldclass athletes. WU content was classified as maximal if it met the “RAMP” criteria [53]; otherwise, it was considered submaximal. These classifications were derived from the original descriptions in the included studies. If participant experience levels were not detailed, they were assigned to the lowest level (i.e., “0.”).

The age of participants was analyzed based on the mean values provided in the original studies. When an age range (e.g., 18–35 years) was given, the median value was used. If separate ages for IPC, SHAM, or CON groups were provided, the average age was calculated by summing all ages and dividing by the number of groups. In studies comparing local and remote IPC or other parameter-related comparisons, data were combined (e.g., remote IPC vs. SHAM and local IPC vs. SHAM were merged into IPC vs. SHAM). For studies with incomplete data, we contacted the authors via email to request the full dataset, especially when only post-hoc comparisons were provided without specific pre- and post-test data. For studies with data presented graphically, we used a data extraction tool (WebPlot-Digitizer – Copyright 2010-2024 Ankit Rohatgi) to extract the data. When comparing the number of IPC interventions (e.g., one intervention 48 h before testing, one 24 h before testing, and one 15 min before testing), only the data from the 15 min pre-test intervention (one intervention) were extracted and compared with CON or SHAM data [54].

When extracting time trial data, we encountered studies that provided total distance and speed. In these cases, we calculated the time using the formula: time = distance / speed. Additionally, for studies presenting data as IPC responders and non-responders, or providing individual participant data instead of group data, we extracted the data for each participant and recalculated the mean and standard deviation [55].

### Statistical Analysis

#### Data Synthesis and Effect Measures

In the included studies, two comparison formats were identified:

1) Between-group post-test comparison: in this format, IPC, CON, and SHAM groups lack pre-test data, and only post-test results are directly compared (IPC_post_ vs. CON_post_, IPC_post_ vs. SHAM_post_);
2) Pre-post change value comparison: this format involves groups (IPC, CON, and SHAM) with both pre- and post-test data. The change values for each group are calculated, and differences in change magnitudes between groups are then compared.

To establish a unified analytical framework, for the first type of comparison (groups lacking pre-test data), we set the pre-test values to baseline (Mean = 0, SD = 0). We then compared the differences between groups using change values. Additionally, as part of the sensitivity analysis, a comparison model using only post-test data was established to verify the robustness of the study conclusions. The following formulas were used to calculate change values an ES:

$${\text{M}}_{\text{change}}={\text{M}}_{\text{post}}-{\text{M}}_{\text{pre}}$$

$${\text{SD}}_{\text{change}}={\text{SD}}_{\text{pre}}^{2}+{\text{SD}}_{\text{post}}^{2}-(2\times \text{r}\times {\text{SD}}_{\text{pre}}\times {\text{SD}}_{\text{post}})$$

$$\text{ES}=\sqrt{\frac{\left({\text{M}}_{\text{post}}-{\text{M}}_{\text{pre}}\right)}{{\text{SD}}_{\text{pooled}}}\times (1-\frac{3}{4({\text{n}}_{1}+{\text{n}}_{2}-2)-1})}$$

where M_post_ and M_pre_ are the means of the pre- and post-test performances of the IPC or CON or SHAM; n_1_ and n_2_ are the sample sizes of the pre- and post-test performances of the IPC or CON or SHAM; and SD_pooled_ is the pooled standard deviation of the measurements [56]. The pre-post correlation coefficient *r* was set to 0.6, followed by a sensitivity analysis across the range of 0.5 to 0.9. The ES was calculated as Hedge’s *g*, adjusted for small sample bias.

When standard errors (SE) were reported, SD was calculated using:

$$SD=SE\times \sqrt{N}$$

ES values were classified as *trivial* (0.2), *small* (0.2–0.5), *medium* (0.5–0.8), and *large* (> 0.8) [57].

Heterogeneity was evaluated using *I*^2^, τ^2^, and Q tests, with *I*^2^ of 25%, 50%, and 75% indicating *low, moderate*, and *high* heterogeneity, respectively. The Q test was considered significant at *p* < 0.1. These indicators represent both relative and absolute values of residual heterogeneity, reflecting the amount of unaccounted variability attributed to residual heterogeneity. Prediction intervals (PI) were calculated to account for potential variability in similar future studies.

#### Multi-Level Meta analysis

Given the characteristics of multiple groups and ES in the included IPC studies, a multilevel mixed-effects model was employed for analysis [43–45, 58]. Specifically, the model incorporated study, comparison type (IPC *vs.* CON, IPC *vs.* SHAM), and within-group ES as explicitly nested random intercepts (i.e., within-group ES nested within comparisons, and comparisons nested within studies). The goodness-of-fit of this model was compared to traditional two-level and three-level models, with the final model selected based on the Akaike Information Criterion (AIC) and Bayesian Information Criterion (BIC) criteria [59]. Effects were weighted by inverse sampling variance to account for within-level, comparison-study, and betweenstudy variance [60].

We employed cluster-robust variance estimation [44] methods with small-sample adjustments [61] to account for within-study correlations between ES. Assuming a within-study correlation of 0.6, sensitivity analyses with correlations of 0.4 and 0.8 showed no differences in the meta-analysis outcomes. Model parameters were estimated using the Restricted Maximum Likelihood method. Individual coefficients and their corresponding confidence intervals (CI) were tested using a t-distribution [59]. Three-level meta-analyses were conducted using the *metafor* package in *R* (version 4.3.0; R Core Team, Vienna, Austria) [62], while cluster-robust variance estimation and small-sample adjustments were implemented using the *clubSandwich* package [63, 63].

#### Subgroup and Meta-regression Analysis

Subgroup analyses were conducted on dichotomous moderating variables including sex, comparison type, participants’ training experience, IPC sets, presence of WU, and measurement outcomes to explore their influence on IPC effects. Regression analyses were conducted to determine the optimal IPC timing to enhance physical performance. All models were compared using linear and various non-linear meta-regressions, including simple linear, cubic polynomial (with 2 and 3 knots), restricted cubic spline (with 3 and 4 knots), and natural cubic spline. Furthermore, a range of models were fitted and compared with (1) random intercepts only, (2) a random slope for estimated interval at the study level, and (3) random slopes for estimated interval at both the study and comparison levels to account for potential heterogeneity [64].

In addition to the single-factor regression analysis, multiple linear regressions were conducted with three fixed effects *a priori*: 1) timing, 2) IPC sets, and 3) experience level. A separate regression analysis was also performed to evaluate the influence of WU presence on IPC effects. Model fit was assessed using Akaike Information Criterion and Bayesian Information Criterion. Moreover, we considered real-world applications, as the effects of IPC on athletic performance do not always increase or decrease proportionally with longer or shorter time intervals, making linear regression models unrealistic for evaluating IPC timing [65]. Therefore, we also reported models that better reflect these conditions, even if their goodness of fit was less optimal. The “optim” algorithm was applied to resolve model convergence issues with the control setting *control = list (optimizer = “optim”)*. All regression models were performed using the *metafor* package and visualized with *ggplot2* package [66, 67].

#### Publication Bias and Sensitivity Analysis

Publication bias was assessed using funnel plots [68] combined with Egger’s test [69], with *p* > 0.05 indicating no bias.

Leverage, outliers, and influence were assessed for all meta-analysis models by calculating hat values, Cook’s distances, and studentized residuals, respectively [70–72]. Outliers were flagged if hat values and Cook’s distances exceeded three times the mean, and if studentized residuals were greater than 3. After removing outliers, calculations were re-run. If results remained unchanged, the original model was retained; otherwise, the model excluding outliers was considered [73].

#### Evidence Certainty Assessment

The quality of scientific evidence was evaluated according to the GRADE Handbook [74]. Certainty was rated as *high, moderate, low*, or *very low* using the GRADEpro GDT software. The evaluation considered risk of bias, inconsistency of results, indirectness of evidence, imprecision of results, and publication bias Evidence started at high certainty but was downgraded for: (1) risk of bias: one level for bias, two for serious bias; (2) indirectness: significant heterogeneity without explanation; (3) imprecision: if PICOS criteria were unmet; (4) inconsistency: small sample size or wide confidence intervals; (5) publication bias: downgrade if present [74, 75, 76]. Evidence was upgraded for: (1) large effect sizes; (2) dose-response relationship; (3) plausible residual confounding likely reducing the observed effect [77].

## RESULTS

### Search Results

**FIG. 1:**
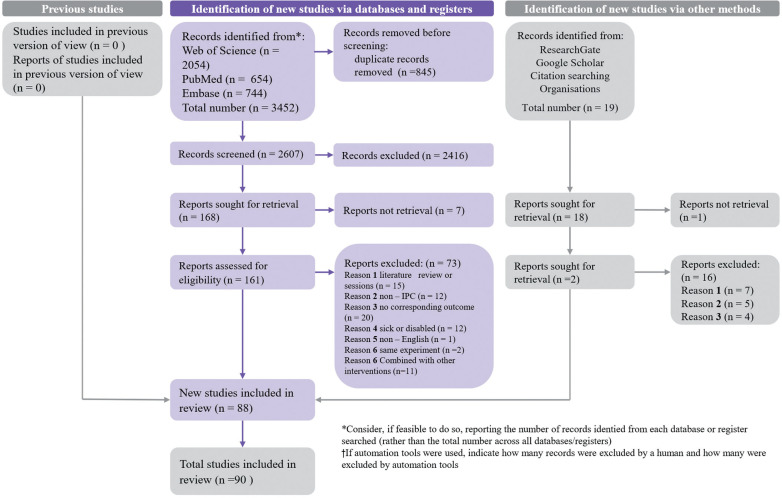
PRISMA flow diagram for included and excluded study.

A total of 3,452 documents were initially retrieved using the developed lead terms. After eliminating duplicates, 2,608 papers were left. Following the screening of titles and abstracts and applying the inclusion and exclusion criteria (Table 1), 88 papers were selected. Two additional papers were included from other sources such as “ResearchGate”, “Google Scholar”, and similar platforms. Therefore, the final number of articles that met the inclusion criteria was 90 (Fig. 1).

### Study Characteristics

Supplementary Material provides a detailed list of the study characteristics for the 90 included articles [10–26, 28, 29, 31, 32, 34–38, 40, 55, 78–139], encompassing demographic factors and IPC-related parameters. All 90 articles were acute randomized crossover (85 studies) or randomized controlled trials (between-participants design; 5 studies), involving a total of 1,439 participants, of whom 1,033 were males and 210 were females, with 196 had unspecified sex. Based on exercise duration of 75 s, 46 articles examined anaerobic exercise [10–15, 22, 23, 25, 26, 28, 29, 40, 80–112], while 41 articles focused on aerobic exercise [16–21, 31, 32, 34–38, 55, 113–139]. Three articles reported outcomes for both anaerobic and aerobic exercise [24, 78, 79]. Additionally, 20 articles did not include a WU protocol.

### Risk of Bias

The physical performance risk of bias for all selected studies was assessed as “some concerns.” According to the RoB 2.0 tool, the primary reasons were: (1) failure to report baseline characteristics of participants; and (2) incomplete participation, leading to partial missing outcome data (Supplementary Material).

### Risk of Publication Bias and Certainty Assessment

The funnel plot showed a symmetrical distribution, and the Egger test results were non-significant, indicating no publication bias.

For physical performance, anaerobic performance, and aerobic performance, no downgrading was applied given the absence of publication bias, high heterogeneity, and consistency with the PICOS framework. However, the quality of evidence was downgraded from high to moderate due to a “some concerns” risk of bias. Conversely, evidence for physical performance was upgraded back to *high*, ES demonstrated a dose–response relationship influenced by warm-up, interval timing, sham, and outcome measurements.

Across different outcome measures, ${\dot{\text{V}}\text{O}}_{2}$, time to failure, time to complete, strength, RM, and power output were rated as *low, moderate, very low, low, moderate*, and *moderate*, respectively (Supplementary Material and Table 2).

**TABLE 2:** Certainty Assessment

	Risk of bias	Inconsistency	Indirectness	Imprecision	Publication bias	Certainty	Certainty-Upgraded	Reason for Upgraded
**Effect of IPC on physical performance (anaerobic and aerobic)**	“some concerns”	↓ *I*^2^-2 = 0%; *I*^2^-3 = 9.13%; *I*^2^-4 = 5.74% ↔	Consistent with the PICOS.	↔ Clear direction of effect size ↔	*P*= 0.38 ↔	*moderate*	*high*	Dose-response gradient (WU, interval time and sham)
		
**Effect of IPC on anaerobic**		↓ *I*^2^-2 = 0%; *I*^2^-3 = 13.64%; *I*^2^-4 = 0% ↔		↔ Clear direction of effect size ↔	*P*= 0.33 ↔	*moderate*	*high*	Dose-response gradient (each outcome)
		
**Effect of IPC on aerobic**		↓ *I*^2^-2 = 0%; *I*^2^-3 = 16.53% ↔		↔ Clear direction of effect size ↔	*P*= 0.63 ↔	*moderate*	*high*	Dose-response gradient (each outcome)
		
**Effect of ${\dot{\text{V}}\text{O}}_{2}$ on IPC**		↓ all *I*^2^ <20% ↔		↔ NO clear direction of effect size↓	all *P > 0.05* ↔	*low*	*low*	None
		
**Effect of time to failure on IPC**		↓ *I*^2^ = 49.89% and 0 % ↓		↔ Clear direction of effect size ↔	all *P > 0.05* ↔	*low*	*moderate*	Dose-response gradient (sham)
		
**Effect of time to complete on IPC**		↓ *I*^2^ = 30.91% and 0 %↓		↔ NO clear direction of effect size↓	all *P > 0.05* ↔	*very low*	*very low*	None
		
**Effect of strength on IPC**		↓ all *I*^2^ <20% ↔		↔ NO clear direction of effect size↓	all *P > 0.05* ↔	*low*	*low*	None
		
**Effect of MR on IPC**		↓ *I*^2^-2 = 0.04%; *I*^2^-3 = 25.91%↓		↔ Clear direction of effect size ↔	all *P > 0.05* ↔	*low*	*moderate*	Dose-response gradient (sham)
		
**Effect of power output on IPC**		↓ all *I*^2^ <20% ↔		↔ Clear direction of effect size ↔	all *P > 0.05* ↔	*moderate*	*moderate*	None

↓: demoted one level; ↔: do not demoted level; *I*^2^-*2/3/4*: Heterogeneity of models at different levels;

In addition, for MAOD, jump, and balance outcomes, the sample sizes were less than 10, which was insufficient for Egger’s test and certainty assessment.

### Main Effects

**FIG. 2:**
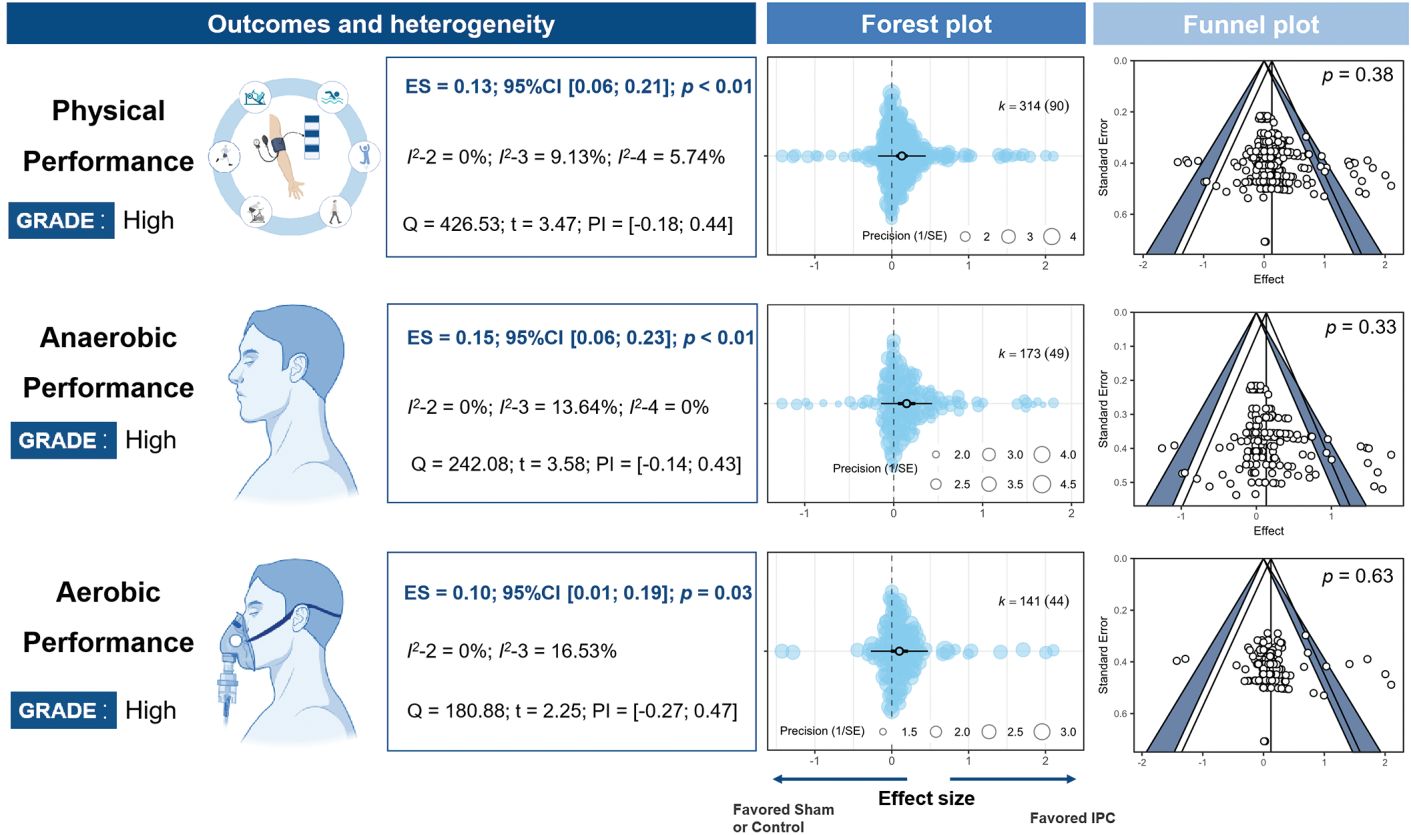
Effects and funnel plot of IPC on performance. Note: ES, effect size; *I*^2^-*2/3/4*, Heterogeneity of models at different levels; *K*, the total number of effects and studies included in the pooled effect size; *P*, statistically significant P values for results; *Q*, Cochran’s Q statistic; SE, standard error; 95% CI, 95% confidence interval; SHAM/CON/IPC, SHAM /Control/IPC Group; PI, prediction interval.

Based on goodness-of-fit comparisons, a four-level model was used to analyze the effects of IPC on physical performance and anaerobic performance, while a three-level model was used for aerobic performance. The results remained consistent across different pre-post correlation (r) values, comparison methods, and after excluding outliers (Supplementary Material). The improvement in physical performance due to IPC was *trivial* (ES = 0.13, 95% CI [0.07; 0.19], *P* < 0.001, *Q* = 427, *I*^2^-Level 2 = 0%, *I*^2^ < -Level 3 = 9.13%, *I*^2^-Level 4 = 5.74%, PI = -0.18; 0.44). Specifically, the effects on anaerobic performance were *trivial* (ES = 0.15, 95% CI [0.05; 0.24], *P* < 0.001, *Q* = 180), as were the effects on aerobic performance (ES = 0.1, 95% CI [0.01; 0.19], *P* = 0.03, *Q* = 242) (Fig. 2). Egger’s tests showed no significant differences (*P* > 0.05). All statistical power values were below 10% (Supplementary Material).

### Subgroup analysis

#### Anaerobic and aerobic performance

The ES of IPC on anaerobic (ES = 0.15) and aerobic performance (ES = 0.10) were comparable, with no significant differences between them (*P* = 0.87, Fig. 2). For anaerobic performance, IPC significantly increased the RM (ES = 0.43, 95% CI [0.12; 0.74], *P* = 0.01). This effect was significantly greater (*P* < 0.05) than improvements in jump performance (ES = 0.05, *P* > 0.05), anaerobic power output (ES = 0.05, *P* > 0.05), strength (ES = 0.06, *P* > 0.05), time trial completion time (ES = 0.11, *P* > 0.05), and ${\dot{\text{V}}\text{O}}_{2}$ (ES = -0.14, *P* > 0.05). For aerobic performance, IPC significantly prolonged time to exhaustion (ES = 0.51, 95% CI [0.16; 0.87], *P* = 0.01), with a smaller but significant improvement in aerobic power output (ES = 0.10, 95% CI [0.00; 0.19], *P* = 0.04). The improvement in exhaustion tests was significantly greater than for other aerobic performance metrics (*P* < 0.05).

#### Comparison

**FIG. 3:**
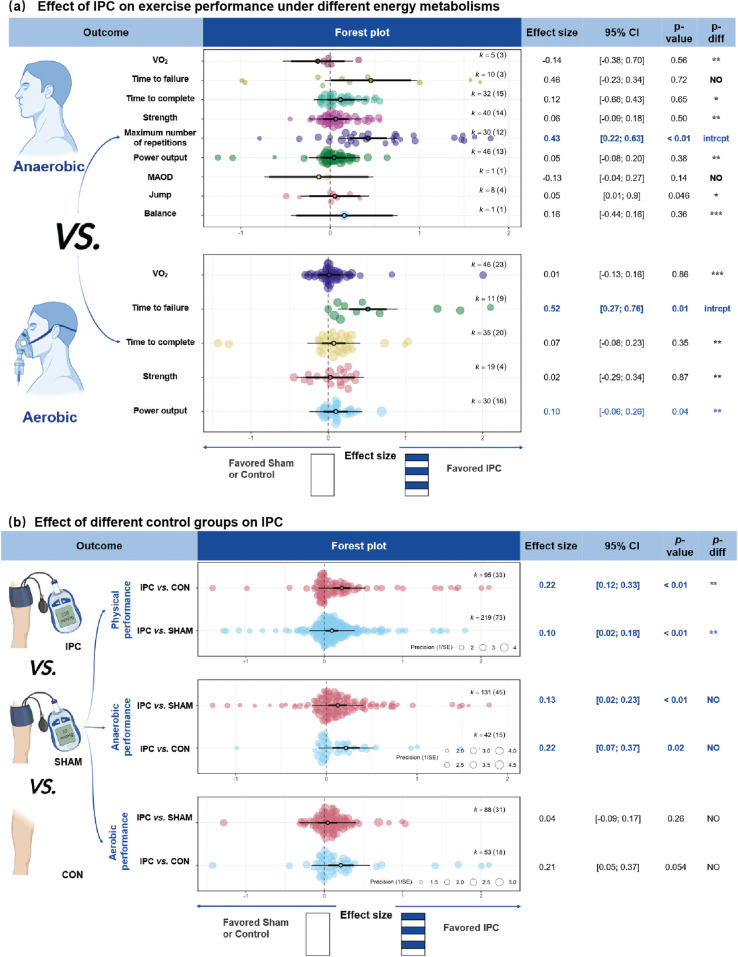
Effects of IPC on performance under different energy metabolisms and different IPC’s control groups. Note: ES, effect size; *K*, the total number of effects and studies included in the pooled effect size; *P*-value, statistically significant *P* values for results; *95%CI*, 95% confidence interval; P, Power Output; RM, Maximum repetitions; *P-diff*, Statistical results of differences between groups; SHAM/CON/IPC, SHAM /Control/IPC Group; MAOD, Maximum acumulative oxygen deficit; ${\dot{\text{V}}\text{O}}_{2}$, oxygen uptake; intrcpt, Comparison with other groups as an intercept; *, Statistically significant and *P-diff* < 0.05 compared to the intercept; **, Statistically significant and *P-diff* < 0.01 compared to the intercept; ***, Statistically significant and *P-diff* < 0.001 compared to the intercept; NO, Statistically insignificant differences between group comparisons and *P* > 0.05.

IPC demonstrated a *small* but significant improvement in physical performance compared to CON (ES of IPC *vs.* CON = 0.22, 95% CI [0.09; 0.35], *P* < 0.01) and a *trivial*, non-significant improvement compared to SHAM (ES = 0.10, 95% CI [0.03; 0.16], *P* = 0.09) (Fig. 3 b). The ES for IPC *vs.* CON was significantly greater than for IPC *vs.* SHAM. (*P* = 0.02). For anaerobic performance, IPC provided a *trivial* but significant benefit compared to both CON (ES of IPC *vs.* CON = 0.18, 95% CI [0.07; 0.30], *P* < 0.01) and SHAM (ES = 0.13, 95% CI [0.04; 0.22], *P* < 0.01). For aerobic performance, IPC showed a *small* improvement compared to CON (ES = 0.22, 95% CI [0.04; 0.40], *P* = 0.02), and a *trivial* improvement compared to SHAM (ES = 0.13, 95% CI [-0.09; 0.17], *P* = 0.01).

Regarding exercise outcome measures, sham effects were generally non-significant (*P* > 0.05). Significant sham effects were found for RM (*P* < 0.01) and time-to-failure tests within aerobic performance (*P* = 0.02; Table 3). Sham effect analyses could not be conducted for other outcomes due to very small sample sizes and the presence of only a single comparison group; for example, jump performance was assessed only in IPC vs. SHAM comparisons.

**TABLE 3:** Results of sham effects across different outcome measures.

	RM	term	K	n	ES	95%CI	P-value	P-diff
**anaerobic**	**RM**	IPC *vs.* CON	2	4	1.26	[0.69; 1.83]	**< 0.01**	**< 0.01**
		IPC *vs.* SHAM	11	26	0.39	[0.09; 0.69]	**0.01**	

	**strength**	IPC *vs.* CON	4	12	0.16	[-0.10; 0.42]	0.22	0.39
		IPC *vs.* SHAM	13	28	0.06	[-0.14; 0.26]	0.56	

	**Power output**	IPC *vs.* CON	6	14	-0.01	[-0.21; 0.18]	0.89	0.69
		IPC *vs.* SHAM	11	32	0.02	[-0.15; 0.18]	0.82	

	**Time to complete**	IPC *vs.* CON	4	7	0.05	[-0.19; 0.30]	0.39	0.38
		IPC *vs.* SHAM	14	25	0.15	[-0.03; 0.33]	**0.01**	

	**Time to failures**	IPC *vs.* CON	2	5	0.38	[-0.82; 1.57]	0.48	0.86
		IPC *vs.* SHAM	2	5	0.26	[-0.93; 1.46]	0.28	

**aerobic**	${\dot{\text{V}}\text{O}}_{2}$	IPC *vs.* CON	10	21	0.18	[-0.03; 0.39]	0.09	0.18
		IPC *vs.* SHAM	17	25	0.02	[-0.16; 0.20]	0.83	

	**strength**	IPC *vs.* CON	1	2	-0.11	[-0.88; 0.67]	0.78	0.66
		IPC *vs.* SHAM	3	17	0.08	[-0.35; 0.52]	0.69	

	**Power output**	IPC *vs.* CON	7	11	0.00	[-0.26; 0.26]	1.00	0.27
		IPC *vs.* SHAM	11	19	0.15	[-0.05; 0.35]	0.15	

	**Time to complete**	IPC *vs.* CON	9	14	0.11	[-0.18; 0.40]	0.44	0.90
		IPC *vs.* SHAM	14	21	0.09	[-0.15; 0.33]	0.45	

	**Time to failure**	IPC *vs.* CON	5	5	1.17	[0.59; 1.76]	**< 0.01**	0.02
		IPC *vs.* SHAM	5	6	0.13	[-0.43; 0.70]	0.61	

ES, effect size; K, number of studies; n, Total number of effects included; 95%CI, 95% confidence interval; *P*-value, statistically significant *P* values for results; *P-diff*, Statistical results of differences between groups; SHAM/CON/IPC, SHAM /Control/IPC Group; *vs.*: versus;

#### Sex

**FIG. 4:**
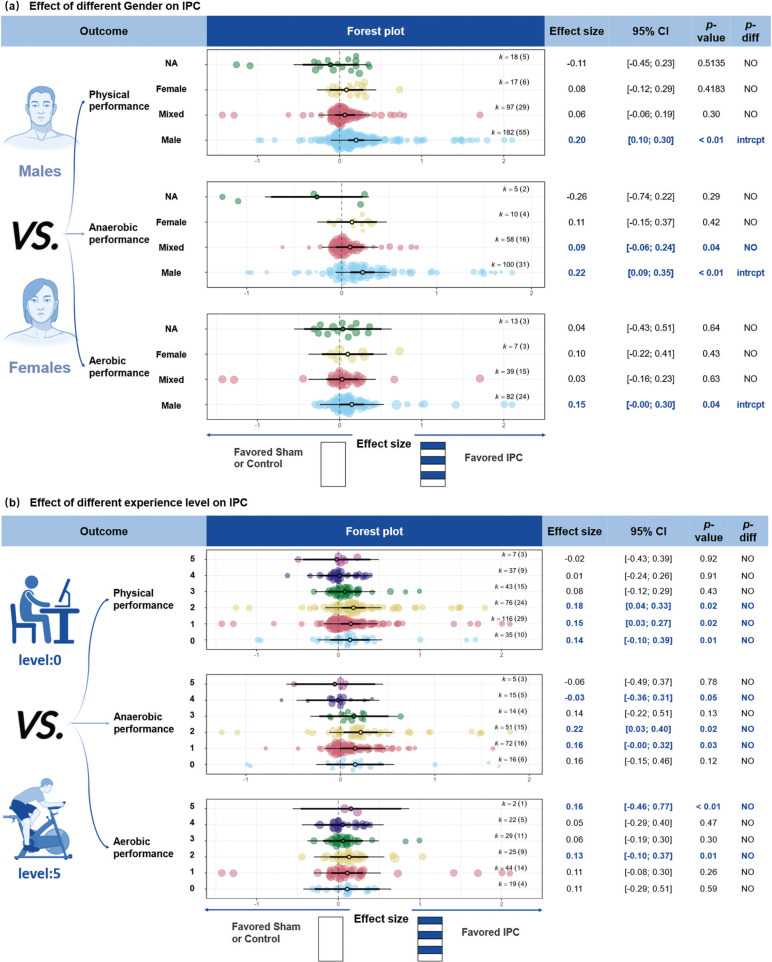
Effect of different Gender and experience level on IPC. Note: ES, effect size; *K*, the total number of effects and studies included in the pooled effect size; *P*-value, statistically significant *P* values for results; *95%CI*, 95% confidence interval; *P-diff*, Statistical results of differences between groups; SHAM/CON/IPC, SHAM /Control/IPC Group; NA, Sex not reported in detail; intrcpt, Comparison with other groups as an intercept; NO, Statistically insignificant differences between group comparisons and *P* > 0.05; 0, sedentary; 1, recreationally active; 2, developing or trained; 3, highly trained or national level; 4, elite or international level; 5, world-class athletes; NO, Statistically insignificant differences between group comparisons and *P* > 0.05.

IPC demonstrated a *small* improvement in physical performance for males (Fig. 4 (a), ES = 0.20, 95% CI [0.12; 0.28], *P* < 0.001), while the effects for females, mixed-sex groups, and groups with unreported sex were not significant (*P* > 0.05). For anaerobic performance, IPC showed a *small* improvement for males (ES = 0.22, 95% CI [0.10; 0.35], *P* < 0.001) and a *trivial* improvement in mixed-sex participants (ES = 0.09, 95% CI [0.01; 0.17], *P* = 0.04). There were no significant differences between sex groups (*P* > 0.05). For aerobic performance, IPC showed a *trivial* improvement in males (ES = 0.15, 95% CI [0.01; 0.29], *P* = 0.04), while the effects for females, mixed-sex groups, and groups with unreported sex were not significant.

#### Training Experience Level

IPC showed a *trivial* improvement in physical performance across participant training levels: *sedentary* (Fig. 4 (b), ES = 0.14, 95% CI [0.02; 0.26], *P* = 0.02), *recreationally active* (ES = 0.15, 95% CI [0.02; 0.27], *P* = 0.02), and *developing or trained* participants (ES = 0.18, 95% CI [0.04; 0.33], *P* = 0.01). For anaerobic performance, a *trivial* improvement was observed in *recreationally active* participants (ES = 0.16, 95% CI [0.02; 0.29], *P* = 0.03). For aerobic performance, *trivial* improvements were seen in *sedentary* (ES = 0.11, 95% CI [-0.02; 0.24], *P* = 0.07), *developing or trained* (ES = 0.13, 95% CI [0.0389; 0.2255], *P* = 0.01), and *world-class* athletes (ES = 0.16, 95% CI [0.14; 0.17], *P* < 0.01).

#### Ischemia and reperfusion time

**FIG. 5:**
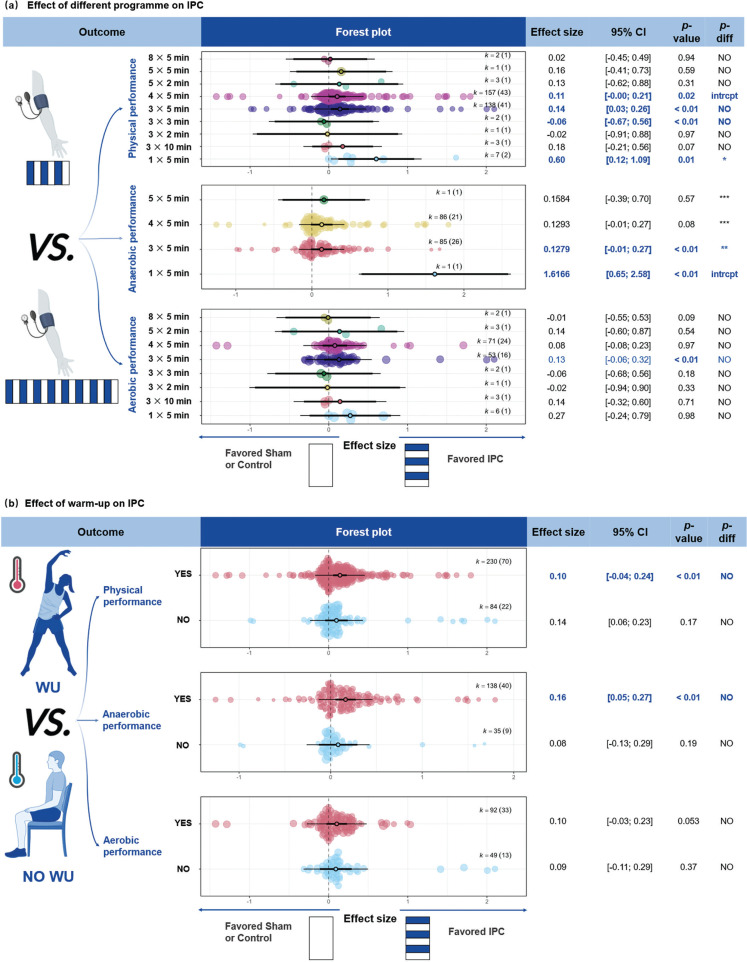
Effect of warm-up and IPC programme on performance. Note: ES, effect size; *K*, the total number of effects and studies included in the pooled effect size; *P*-value, statistically significant *P* values for results; *95%CI*, 95% confidence interval; *P-diff*, Statistical results of differences between groups; SHAM/Control/IPC, SHAM /Control/IPC Group; min, minute; intrcpt, Comparison with other groups as an intercept; *, Statistically significant and *P-diff* < 0.05 compared to the intercept; **, Statistically significant and *P-diff* < 0.01 compared to the intercept; ***, Statistically significant and *P-diff* < 0.001 compared to the intercept; NO, Statistically insignificant differences between group comparisons and *P* > 0.05.

Ischemia-reperfusion protocols of 4 × 5-min (ES = 0.10, 95% CI [0.02; 0.19], *P* = 0.02) and 3 × 5-min (ES = 0.14, 95% CI [0.03; 0.25], *P* < 0.01) were found to enhance physical performance (Fig. 5 (a)). For anaerobic efforts, both the 1 × 5-min (ES = 1.62, 95% CI [0.65; 2.58], *P* < 0.01) and 3 × 5-min (ES = 0.13, 95% CI [0.04; 0.21], *P* < 0.01) protocols significantly improved performance. Significant differences were observed between the 1 × 5 min protocol and the 3 × 5-min, 4 × 5-min, and 5 × 5-min protocols (*P* < 0.01). For aerobic performance, ischemia-reperfusion durations of 1 × 5-min (ES = 0.27, 95% CI [-0.24; 0.78], *P* = 0.09) and 4 × 5-min (ES = 0.08, 95% CI [-0.01; 0.16], P = 0.09) tended to improve aerobic performance.

#### WU and IPC

IPC combined with WU significantly enhanced physical performance (Fig. 5 (b), ES = 0.14, 95% CI [0.07; 0.22], *P* < 0.01), whereas no significant effect was observed without WU (ES = 0.10, 95% CI [-0.08; 0.27], *P* = 0.25). For anaerobic performance, IPC combined with WU showed a significant improvement (ES = 0.16, 95% CI [0.06; 0.26], *P* < 0.01), while no significant effect was observed without WU (ES = 0.08, 95% CI [-0.06; 0.22], *P* = 0.19). For aerobic performance, neither the presence (*P* = 0.052) nor absence (P = 0.54) of WU showed significant improvements.

### Meta-regression analysis

**FIG. 6:**
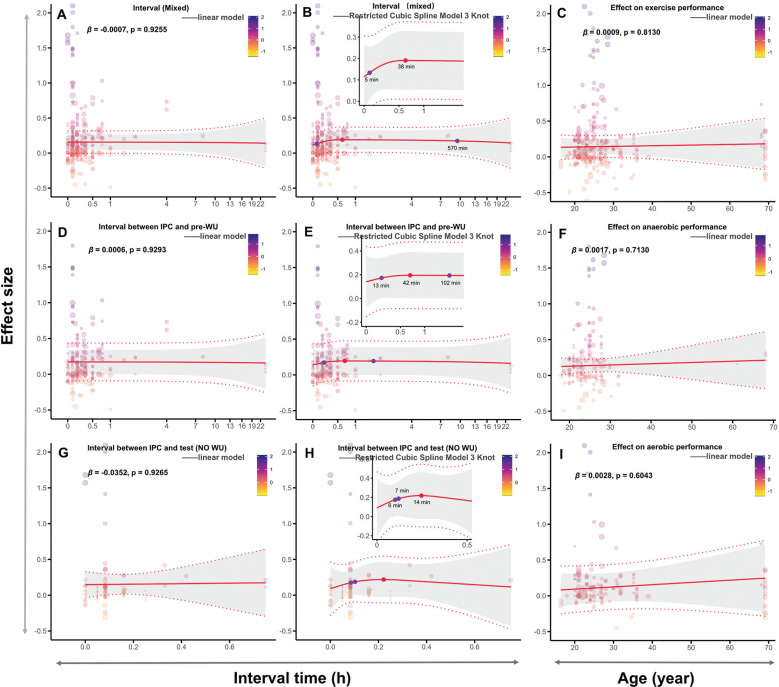
Meta-Regression analysis: effect of interval and age on IPC. Note: Mixed, both with and without warm-up; WU, warm-up; pre-WU, before the warm-up; h, hour; min, minute; β, regression coefficient; *P*, statistically significant *P* values for results; A and B, linear and nonlinear regression analyses of the effect of the interval between IPC and testing on physical performance, without considering the WU protocol; D and E, linear and nonlinear regression analysis of the effect of the interval between IPC and WU on physical performance; G and H, linear and nonlinear regression analysis of the effect of the interval between IPC and testing on physical performance in the absence of WU; C, F and I, linear regression analyses of age and IPC on overall performance, anaerobic performance, and aerobic performance, respectively.

Among all fitted models, the linear meta-regression for the singlefactor interval demonstrated the best fit. However, non-linear regression models better explained the timing of IPC’s effects on performance, capturing the non-monotonic relationship over time. The interval from IPC to testing ranged from 5 to 570 min, with the optimal improvement at approximately 37 min (Fig. 6 B). With WU, the interval from IPC to performance testing ranged from 13 to 102 min, with the optimal effect at 42 min (Fig. 6 E). Without WU, the IPC-to-testing interval ranged from 6 to 7 min, with the optimal interval at 14 min, although this time point was not statistically significant (Fig. 6 H, P > 0.05). Age did not significantly predict or moderate the performance-enhancing effects of IPC. Detailed analysis results can be found in Supplementary Material.

## DISCUSSION

This systematic review and meta-analysis assessed the effects of IPC on physical performance, identified the optimal timing, and explored the influence of moderating factors including sex, training level, ischemia-reperfusion duration, and the presence of WU. The main findings indicated IPC had a *trivial* effect on overall physical performance, with variations based on exercise type and testing conditions. For anaerobic performance, IPC significantly improved RM with a *small* ES, while no significant improvements were observed for jump performance, anaerobic power output, strength, time-trial completion time, or ${\dot{\text{V}}\text{O}}_{2}$. Regarding aerobic performance, IPC significantly prolonged time to exhaustion with a *medium* ES, outperforming other aerobic outcomes. Additionally, IPC produced *small* to *trivial* improvements in physical performance compared to both CON and SHAM groups, with a more pronounced effect relative to SHAM, indicating a placebo component. However, performance benefits persisted after accounting for placebo effects, supporting the efficacy of IPC.

IPC demonstrated larger improvements in physical performance for males, particularly in anaerobic exercises, while non-significant effects were noted for females, mixed-sex groups, or groups with unreported sex. *Sedentary, recreationally active* and *developing or trained* participants experienced only *trivial* improvements. Furthermore, IPC effectiveness was influenced by protocol duration, with 4 × 5-min and 3 × 5-min protocols showing the greatest enhancements in physical performance. Anaerobic performance significantly improved with 1 × 5-min and 3 × 5-min protocols.

When combined with a WU, IPC showed larger improvements in physical performance, particularly in anaerobic performance. However, IPC alone, without WU, had no significant effects. The optimal timing for IPC was approximately 37 min before exercise testing (regardless of WU), with the most notable effects observed at 42 min when paired with WU, and an effective range of 6-7-min without WU. Age did not significantly influence the effects of IPC.

## Effects of IPC on Physical performance and WU Protocols

This study demonstrates that IPC can boost physical performance (ES = 0.13), with *trivial* but significant effects on both anaerobic (ES = 0.14) and aerobic performance (ES = 0.10). However, there was no significant difference in the magnitude of improvement between anaerobic and aerobic performance. These findings align with those of Salvador et al. [140] and Marocolo et al. [141], who also reported positive effects of IPC on both types of performance. Additionally, IPC was particularly effective in improving exercise endurance, with significant increases in time to exhaustion (ES = 0.52) and the RM (ES = 0.43), both showing larger ES compared to other performance metrics (Fig. 3). These results are consistent with a recent metaanalysis [39], which found significantly enhancements in anaerobic endurance (mean difference, MD = 0.68) and time to exhaustion in aerobic exercise (MD = 22.68).

From a mechanistic standpoint, IPC may enhance endurance capacity by increasing nitric oxide (NO) and adenosine concentrations, activating K-ATP channels, and improving mitochondrial function [142, 143]. It may also enhance endurance performance by improving hypoxia tolerance [139] and modulating perceived exertion [20]. Nonetheless, Sabino-Carvalho et al. [132] found that IPC-induced endurance improvements may largely reflect placebo effects, as gains did not significantly exceed those in the sham group. This finding aligns closely with our study. In aerobic endurance tasks (time-to-failure tests), IPC improved performance compared with CON, but no significant benefit remained after accounting for sham effects (Table 3). Given the *trivial* ES, placebo likely plays a key role in IPC’s influence on endurance performance. Neural regulation independent of metabolic changes – such as reduced inhibition of central motor drive via suppression of type III/IV muscle afferent feedback – may also play a role in determining endurance limits [144, 145]. Supporting this, IPC has been shown to improve neuromuscular recovery, such as reducing force decline during repeated isometric contractions [146]. Further evidence suggests IPC may reduce presynaptic inhibition, increase motor neuron excitability, and modulate sympathetic nervous system activity [46, 87]. For example, IPC can enhance resistance performance by increasing neural excitability, thereby inducing a higher level of arousal [147]. Therefore, IPC likely enhances endurance through a multifactorial synergy involving metabolic pathways (NO activation, mitochondrial efficiency, hypoxia tolerance), neural mechanisms (reduced central motor inhibition, sympathetic modulation), and potential placebo effects. This underscores the complex, multidimensional nature of IPC’s mechanisms and highlights the need for further research to disentangle the contributions of each component.

From a study design perspective, we propose that IPC may have an additive effect when combined with a WU. For example, one study found no improvement in 50-meter sprint swimming performance 1 h after IPC, but significant benefits at the 2-h and 8-h marks [40]. However, this study added a 20-min WU within the 1-h, 2-h, and 8-h intervals after IPC, meaning the interval was actually measured from IPC to the start of WU, not from IPC to the testing itself [40]. Similar issues were observed in studies by Valenzuela et al. [29] and Richard et al. [25] Additionally, some studies applied IPC between WU and testing phases [106, 111, 137]. The inconsistent definitions of IPC-to-testing intervals, along with heterogeneity in intervention timing (e.g., pre- or post-warm-up), may obscure IPC’s independent effect on athletic performance, compromising the reliability of estimates on its true impact in current meta-analyses and experimental research.

Among the studies included in our analysis, 20 did not include WU protocols, while 70 did. Subgroup analyses revealed that adding WU after IPC significantly improved physical performance (ES = 0.14, *P* < 0.01), while no significant effect was observed without WU (*P* = 0.13) (Fig. 5 (b)). Numerous studies have demonstrated that WU enhances short-, medium-, and long-term physical performance [148–153], with varying effects depending on the specific WU strategy [148, 152]. However, no studies have directly investigated whether IPC and WU have additive effects, though some have indirectly addressed this. For example, Vangsoe et al. [112] compared IPC, post-activation potentiation, and self-selected WU strategies on 1-km time trial performance and found no significant differences among the three. Additionally, Guilherme da Silva Telles et al. [93] compared the effects of IPC, SHAM IPC, specific WU, aerobic exercise, and active stretching on resistance training outcomes, and found that IPC combined with resistance training significantly increased RM and total training volume.

In summary, the crucial role of IPC in enhancing physical performance may have been overlooked, contributing to the variability in its effects. Future guidelines for research end-users should propose to standardize IPC protocols – including well-defined WU procedures and precise interval timing – to minimize heterogeneity. Additionally, rigorous experimental designs are needed to isolate IPC’s true physiological effects from sham influences, particularly under controlled conditions (e.g., standardized WU and intervals).

## IPC Interval Timing Based on WU Control

This meta-analysis found that, compared to traditional cardioprotective protocols in animals (early and late phases, typically 2–3 or 12–24 h) [142, 154–156], the effective IPC protocol to boost performance in humans is significantly shorter. Specifically, the optimal interval for IPC to enhance physical performance (regardless of WU) was approximately 38 min (range: 5 to 570 min). With WU, the optimal interval from IPC to the start of WU extended to 42 min (range: 13 to 102 min). Without WU, the effective interval time was reduced to 6–7 min. These findings align with Incognito et al. [2]‘s systematic review, which suggested that variability in IPC effects on physical performance is primarily due to differences in interval times.

Although our results differ from the traditional early (2–3-h) and late (12–24-h) IPC-induced endothelial protection windows [142, 154–156], this discrepancy may stem from the fact that classical studies focused on animal models and did not consider exercise interventions. However, our findings partly align with Lindner et al. [26], who compared the effects of IPC with 0-min or 20-min intervals before WU on strength and sprint performance. They found no significant differences but recommended a 25–45-min interval from IPC to WU for optimal performance. Additionally, Seeley et al. [157] demonstrated that a 45-min interval from IPC to WU improved muscle oxygen saturation in the recovery phase after sprint exercise, outperforming a 5-min interval. These conclusions align closely with our finding of a 42 min optimal interval, further supporting the notion that combining IPC with WU extends its physiological benefits.

Mechanistically, IPC may enhance endothelial nitric oxide synthase (eNOS) activity, promoting NO release and thereby improving vasodilation and oxygen delivery [158]. Exercise itself also activates eNOS through shear stress, increasing NO synthesis and consumption [159]. The release of NO typically peaks within minutes and gradually returns to baseline levels [160]. Without a WU, the physiological benefits of NO may not last long, which may explain the short effective interval for IPC (6–7 min). In contrast, when WU is included, the optimal interval extends to 42 min (range: 6 to 102 min). This may occur because IPC activates eNOS to release NO, while WU induces a secondary NO release (via adenosine receptor signaling) combined with the increase in body temperature. This synergistic effect enhances hemodynamic responses [161] and allows the benefits of IPC to extend into the testing phase.

From a practical perspective, experimental designs without WU (6–7-min interval) may be better suited for mechanistic studies of IPC, as they offer better control over variables. However, in real-world applications such as competitive settings, it is recommended to perform IPC before a WU (42-min interval) to avoid potential injury risks associated with high-intensity exercise. Additionally, low-intensity exercise can induce physiological benefits associated from IPC. For example, Telles et al. [110] found that both low and high-pressure IPC similarly enhance 1 RM testing through more favorable psychophysiological responses, such as neural stimulation of type III and IV afferent fibers. Additionally, IPC combined with varying-intensity exercise (e.g., 50% MVC grip training [162], 5-km time trials [16], and exhaustion tests at 70% ${\dot{\text{V}}\text{O}}_{2\max}$ [122, 123]) can effectively improve physical performance or physiological indicators.

## Is the Potent Effect of IPC Derived from SHAM?

This study found that the performance-enhancing effects of IPC are partly influenced by SHAM effects. Specifically, IPC significantly improved physical performance compared to CON (ES = 0.13, *P* < 0.01), and this effect was significantly greater than the effect observed with SHAM (*P* = 0.02). In addition, IPC produced large improvements in the maximum number of repetitions (RM, ES = 1.26) and time to failure (ES = 1.17) compared to CON, which were also significantly greater than those in the SHAM group (Table 3). These findings are supported by subgroup analyses, where the effect size for time to failure (ES = 0.52) was significantly greater than that for other outcomes (Fig. 3a), with RM showing a similar pattern. However, in aerobic performance, the time to failure test did not show significant improvement after adjusting for sham effects. This suggests that the performanceenhancing effects of IPC may be overestimated without proper sham controls. This interpretation aligns with previous meta-analyses showing IPC improves performance versus control, while SHAM group also outperforms CON [41, 42]. Likewise, de Souza et al. [28] found no additional performance IPC benefits in resistance exercise, likely due to sham effects, and Santos Cerqueira et al. [163] suggested that the muscle-protective effects of IPC following eccentric exercise may improve placebo responses.

The SHAM effect may originate from the potential influence of IPC on sensory nerves. For example, Marocolo et al. [9] found that both real IPC and sham interventions induced tactile sensations from ischemic pressure, stimulating subcutaneous low-threshold mechanoreceptors (e.g., Pacinian corpuscles, Meissner corpuscles, Merkel discs, and Ruffini endings) and modulating neural processing. This suggests that IPC might elicit positive expectations in participants, ultimately enhancing overall performance. Supporting this, Souza et al. [164] demonstrated that verbal instructions manipulating expectations in a sham group significantly affected resistance performance, with participants holding positive expectations performing better than those with negative or neutral expectations. Although sham effects contribute to IPC’s performance benefits, they may not fully explain its efficacy. Even after controlling for sham effects (IPC vs. SHAM), IPC still improved physical performance (ES = 0.10, *P* < 0.01; Fig. 3b), a pattern also evident in anaerobic outcomes such as RM and time to completion (Table 3). In addition, the studies reviewed revealed significant variability in IPC interval times (0–60 min), with four studies exceeding 60 min [41, 42]. Additionally, these studies did not account for the inclusion of a WU, which is known to enhance physical performance [148–153]. Our metaanalysis showed that adding WU after IPC significantly improved physical performance (ES = 0.14, *P* < 0.01), whereas no significant effect was observed without WU (P = 0.125).

Future research should aim to isolate the true physiological mechanisms of IPC by strictly controlling for variables like WU, interval timing, and verbal instructions to determine whether observed benefits are genuinely independent of sham effects.

## Ischemia-Reperfusion Duration

This study found that ischemia-reperfusion protocols of 4 × 5 min (ES = 0.10, *P* = 0.02) and 3 × 5-min (ES = 0.14, *P* < 0.01) significantly improved physical performance. These findings align with previous research indicating that 3 × 5-min or 4 × 5-min IPC protocols are effective to induce the desired physiological stimuli [5–8, 165].

In addition to the 3 × 5-min and 4 × 5-min IPC protocols, some studies have employed other ischemia-reperfusion cycles, such as 8 × 5-min [34], 5 × 5-min [108], and 5 × 2-min [126], as well as longer [136] or shorter [137] ischemia durations. However, in our analysis of anaerobic performance, a very *large* ES was observed for the shorter IPC protocol (1 × 5-min; ES = 1.62), but this result should be interpreted with caution due to data from only a single study. Interestingly, Gkari et al. [166] reported that a very short IPC duration (1 × 5-min) significantly improved performance in track and field high jump athletes as well as vertical jump outcomes. Therefore, such an ultra-short IPC protocol may be a time-efficient and potentially effective strategy for performance enhancement. In a study by Cocking et al. [34], the ultra-long IPC protocol (8 × 5-min) did not improve cycling time trial performance, while the 4 × 5-min protocol did. Based on current evidence, we recommend using 3 × 5-min or 4 × 5-min protocols to optimize physical performance.

## Training Experience Level

IPC did not show significant effects on physical performance in participants with higher training levels (≥ *highly trained or national level* participants), according to the performance caliber classification of McKay et al. [52] (*levels 0-5*). However, a significant effect on aerobic performance was observed in participants at world-class athletes. It is important to note that this result may not be fully representative, as only one study included participants at this level.

IPC appears to be more effective for individuals with lower training experience (*sedentary, recreationally active* and *developing or trained* participants) (Fig. 4 (b)). This may be because individuals with lower training levels have more room for improvement, while those with higher levels may be closer to their genetic potential [39]. For example, one study found no significant difference at well-trained runners in 5-km performance improvement when IPC was applied 24-h before WU *versus* immediately before, suggesting that training experience and exercise type influenced the results [31]. Previous meta-analyses categorized participants as trained or untrained and found differences only in ratings of perceived exertion [41]. However, they did not explore the sources of these differences. We argue that this classification may not be universally applicable, as significant variations in training levels exist within both groups.

In the analyses for anaerobic (*P* = 0.12) and aerobic (*P* = 0.07) efforts, IPC showed no additional performance benefits for sedentary individuals (*sedentary*) (Fig. 4 (b)). While it is easier to identify the upper limits of physiological abilities in athletes, determining the lower limits in sedentary populations remains difficult. Even within sedentary groups, genetic factors may provide some individuals with inherent advantages. For example, in standardized aerobic training, individual variations in ${\dot{\text{V}}\text{O}}_{2\max}$ gains are substantial, with approximately 50% of the variation attributed to genetics [167]. Additionally, studies on monozygotic twins have shown that environmental factors can cause notable differences in training levels and body weight, with exercise also inducing epigenetic changes that lead to different health outcomes [168]. This suggests that environmental factors can significantly affect training levels, even among those who are sedentary.

## Sex and age

We found that sex does not significantly moderate the performanceenhancing effects of IPC. However, males derived greater performance benefits from IPC, with notable improvements in physical performance physical performance, as well as aerobic and anaerobic performance (Fig. 4 (a)). In support, a sex-specific study found that IPC enhanced time to task failure only in males [15]. Other research suggests that IPC may influence functional sympathetic modulation, thereby improving grip strength, but this effect was observed exclusively in males [46]. It was proposed that the enhanced endurance [15] and improved grip strength performance [46] in males following IPC may result from greater ${\dot{\text{V}}\text{O}}_{2}$ and strength gains. Nonetheless, the influence of sham effects, WU protocols, and interval timing cannot be ruled out. For instance, despite the better performance outcomes in males, Pereira et al. [15] speculated that individual differences in pain perception might play a crucial role in explaining these results.

Although our regression analysis found that age does not moderate IPC’s performance-enhancing effects, most participants in our study were aged 20–30 years, with few participants aged 60–70 years, and none in the 40–60 age range (Fig. 6 C, Fig. 6 F, Fig. 6 I). This uneven distribution of age groups may have influenced the results. Standalone IPC (without exercise) seem ineffective for promoting health in older adults. For example, a study comparing high-intensity interval training, isometric handgrip training, and IPC found no significant reduction in systolic blood pressure reduction with IPC [169]. However, when IPC is combined with exercise, it may still offer benefits for older adults. Research has shown that IPC can acutely improve handgrip strength and functional capacity in active older women [79], enhance balance [108], and reduce systolic blood pressure in older adults without causing additional fatigue [130]. These findings highlight that IPC could serve as a time-efficient health promotion strategy for older adults.

## Certainty of Evidence and Risk of Bias

Using the GRADE framework, the certainty of evidence for the overall effects of IPC on physical performance ranged from *moderate* to *high*, particularly for endurance-related outcomes such as time to exhaustion and RM, where dose–response relationships (e.g., warmup integration, interval timing, and sham controls) supported upgrading. Conversely, the certainty of evidence for ${\dot{\text{V}}\text{O}}_{2}$, strength, and time-to-completion outcomes remained *low* to *very low* due to imprecision and inconsistent ES. These results suggest that while IPC demonstrates small but significant ergogenic benefits overall, the strength of evidence varies considerably across outcome measures.

Risk of bias assessments indicated “some concerns” in most included studies. The primary sources of bias included small sample sizes, insufficient randomization and allocation concealment, and the inherent difficulty of blinding participants and researchers in perceptible IPC interventions. Additional concerns included selective reporting and incomplete descriptions of intervention protocols. The observed sham effects further underscore the challenges of adequately blinding participants in IPC research.

Taken together, although moderate-to-high certainty supports IPC’s benefits for endurance-related outcomes, the low certainty for other measures warrants cautious interpretation.

### Practical Applications

This study addresses existing controversies surrounding IPC and proposes feasible, standardized protocols to optimize its performanceenhancing effects. Based on our meta-analytic findings, we recommend two standardized IPC protocols suitable for experimental or applied contexts (see Conclusion section).

Evidence indicates that IPC can improve physical performance (ES = 0.13), though the effect may be relatively *trivial* and partly influenced by sham. However, sham effects should not be viewed strictly as a limitation, since they may contribute to IPC’s ergogenic potential. When IPC provides immediate performance benefits, its application is justified. Notably, IPC produces substantial improvements in endurance-related outcomes, such as time-to-failure in aerobic exercise and the maximal number of repetitions in anaerobic exercise. These findings support the practical value of IPC as an acute performance-enhancing strategy, particularly in resistance and aerobic activities.

Beyond acute performance enhancement, emerging evidence suggests that IPC may also support training recovery and load management. Applying IPC before or after strength training has been shown to facilitate post-exercise recovery and reduce fatigue compared with traditional recovery methods such as static stretching or foam rolling [170]. Moreover, combining IPC with palm cooling during highintensity resistance training further enhances performance by augmenting arousal responses [147]. These findings suggest that IPC can serve not only as an acute ergogenic aid but also as a complementary tool for recovery optimization and integrated performance strategies in both athletic and rehabilitation contexts.

## CONCLUSIONS

IPC can significantly enhance physical performance measures (e.g., RM, time to failure, power output, and jump), supported by *moderate* to *high* certainty of evidence. However, it appears ineffective for ${\dot{\text{V}}\text{O}}_{2}$, strength, time to completion, MAOD, and balance, where evidence certainty is *low* to *very low*. Its effects are strongly influenced by sham conditions, warm-up protocols, and the specific IPC protocol applied. Moreover, most studies presented “some concerns” or “high” risk of bias, highlighting methodological and reporting limitations in the current literature.

The most effective approach appears to be 3–4 × 5-min ischemia with an interval of approximately 42 min, followed by a standardized warm-up and endurance testing (e.g., time to exhaustion). This strategy shows the greatest benefits in males with lower training levels. For mechanistic studies, warm-up should be excluded and the IPC-to-testing interval limited to 6–7 min to better isolate physiological effects. Researchers should also clearly define whether “interval” refers to IPC-to-WU or IPC-to-testing to avoid ambiguity. The potential benefits of IPC for women, middle-aged populations, and elite athletes warrant further investigation.

## Supplementary Material

Enhancing physical performance with ischemic preconditioning: a systematic review and meta-analysis of moderators and performance outcomes

## Data Availability

All data, codes and analysis process are available at https://github.com/Zhangyilin-coder/IPC.
