# Supplementary material for: Enhancing physical performance with ischemic preconditioning: a systematic review and meta-analysis of moderators and performance outcomes
Source: Biol Sport. 2025 Oct 31;43:511–54. doi: 10.5114/biolsport.2026.154945 (PMC12954497; doi:10.5114/biolsport.2026.154945)
Supplement: Enhancing physical performance with ischemic preconditioning: a systematic review and meta-analysis of moderators and performance outcomes [file JBS-43-56933-s1.pdf]

SUPPLEMENTARY

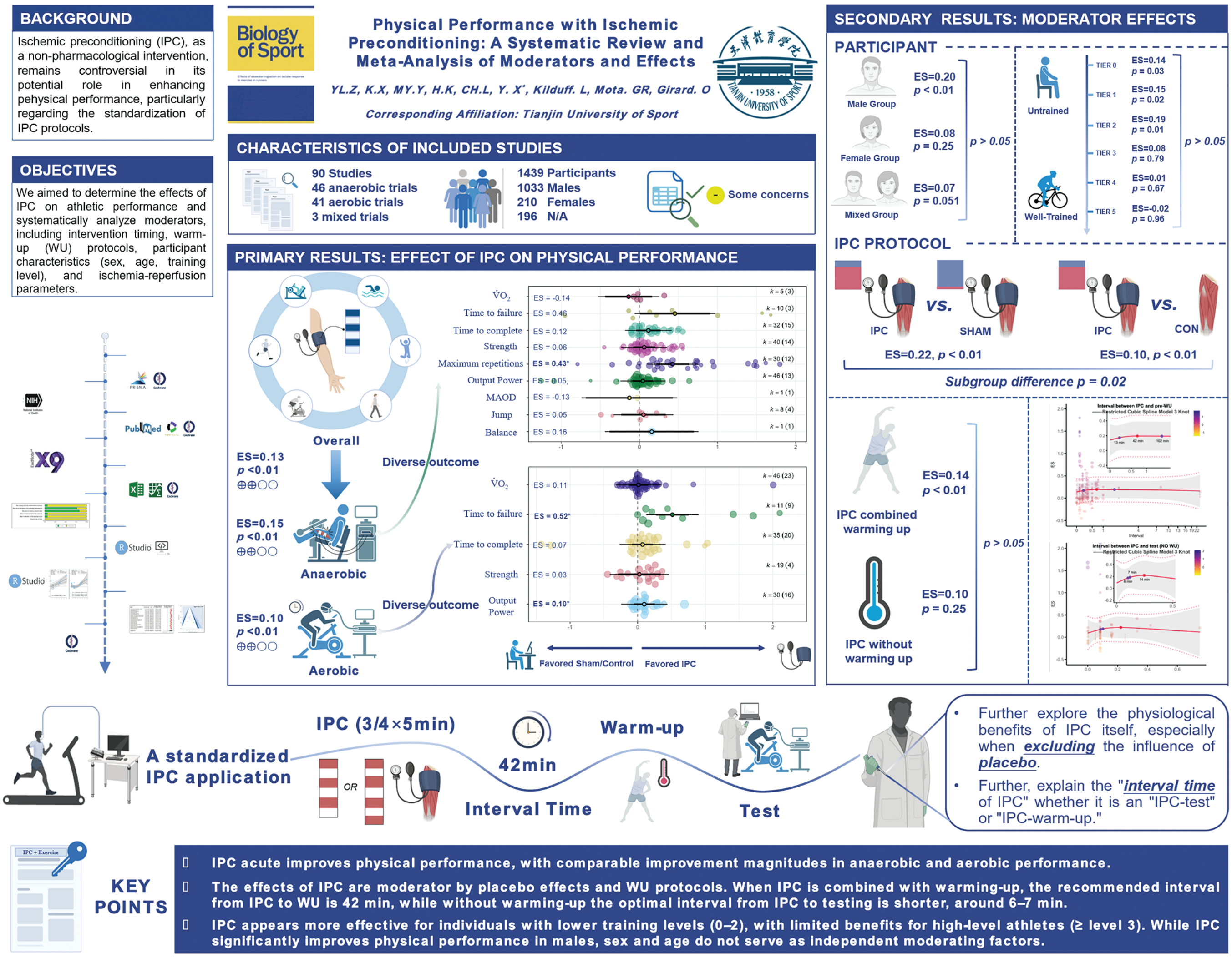

FIG S1. Abstract

ELECTRONIC SUPPLEMENTARY MATERIAL CONTENTS

TABLE S1.

Number	Material	Page
1	Electronic Supplementary Material Appendix S1 (PRISMA 2020 checklist)	
2	Electronic Supplementary Material Appendix S2 (Search strategy)	
3	Electronic Supplementary Material Appendix S3 (Physical performance risk of bias assessment)	
4	Electronic Supplementary Material Appendix S4 (Characteristics of the included studies)	
5	Electronic Supplementary Material Appendix S5 (Goodness-of-fit and correlation coefficient comparison results)	
6	Electronic Supplementary Material Appendix S6 (Statistical power analysis)	
7	Electronic Supplementary Material Appendix S7 (Physical performance detailed results of data analysis	
8	Electronic Supplementary Material Appendix S8 (Funnel plots with Egger's test for exercise outcomes across different energy systems)	

ELECTRONIC SUPPLEMENTARY MATERIAL APPENDIX S1 (PRISMA 2020 CHECKLIST)

TABLE S2.

Section/topic	#	Checklist item	Reported on page #
Enhancing Physical Performance with Ischemic Preconditioning: A Systematic Review and Meta-Analysis of Moderators and Performance Outcomes			
Title	1	Identify the report as a systematic review, meta-analysis, or both.	1
Structured summary	2	Provide a structured summary including, as applicable: background; objectives; data sources; study eligibility criteria, participants, and interventions; study appraisal and synthesis methods; results; limitations; conclusions and implications of key findings; systematic review registration number.	1
INTRODUCTION			
Rationale	3	Describe the rationale for the review in the context of what is already known.	3, 4, 5, 6
Objectives	4	Provide an explicit statement of questions being addressed with reference to participants, interventions, comparisons, outcomes, and study design (PICOS).	6
METHODS			
Protocol and registration	5	Indicate if a review protocol exists, if and where it can be accessed (e.g., Web address), and, if available, provide registration information including registration number.	6
Eligibility criteria	6	Specify study characteristics (e.g., PICOS, length of follow-up) and report characteristics (e.g., years considered, language, publication status) used as criteria for eligibility, giving rationale.	6, 7
Information sources	7	Describe all information sources (e.g., databases with dates of coverage, contact with study authors to identify additional studies) in the search and date last searched.	7, 8, 9
Search	8	Present full electronic search strategy for at least one database, including any limits used, such that it could be repeated.	6
Study selection	9	State the process for selecting studies (i.e., screening, eligibility, included in systematic review, and, if applicable, included in the meta-analysis).	6, 7
Data collection process	10	Describe method of data extraction from reports (e.g., piloted forms, independently, in duplicate) and any processes for obtaining and confirming data from investigators.	7, 8, 9
Data items	11	List and define all variables for which data were sought (e.g., PICOS, funding sources) and any assumptions and simplifications made.	7, 8, 9
Risk of bias in individual studies	12	Describe methods used for assessing risk of bias of individual studies (including specification of whether this was done at the study or outcome level), and how this information is to be used in any data synthesis.	7
Summary measures	13	State the principal summary measures (e.g., risk ratio, difference in means).	9, 10
Synthesis of results	14	Describe the methods of handling data and combining results of studies, if done, including measures of consistency (e.g., I^2) for each meta-analysis.	9, 10, 11, 12
Risk of bias across studies	15	Specify any assessment of risk of bias that may affect the cumulative evidence (e.g., publication bias, selective reporting within studies).	7
Additional analyses	16	Describe methods of additional analyses (e.g., sensitivity or subgroup analyses, meta-regression), if done, indicating which were pre-specified.	11, 12
RESULTS			
Study selection	17	Give numbers of studies screened, assessed for eligibility, and included in the review, with reasons for exclusions at each stage, ideally with a flow diagram.	11
Study characteristics	18	For each study, present characteristics for which data were extracted (e.g., study size, PICOS, follow-up period) and provide the citations.	12
Risk of bias within studies	19	Present data on risk of bias of each study and, if available, any outcome level assessment (see item 12.	13
Results of individual studies	20	For all outcomes considered (benefits or harms), present, for each study: (a) simple summary data for each intervention group (b) effect estimates and confidence intervals, ideally with a forest plot.	14
Synthesis of results	21	Present results of each meta-analysis done, including confidence intervals and measures of consistency.	14
Risk of bias across studies	22	Present results of any assessment of risk of bias across studies (see Item 15).	13
Additional analysis	23	Give results of additional analyses, if done (e.g., sensitivity or subgroup analyses, meta-regression [see Item 16]).	14, 15, 16, 17

TABLE S2. Continue.

Section/topic	#	Checklist item	Reported on page #
		**DISCUSSION**	
Summary of evidence	24	Summarize the main findings including the strength of evidence for each main outcome; consider their relevance to key groups (e.g., healthcare providers, users, and policy makers).	17, 18
Limitations	25	Discuss limitations at study and outcome level (e.g., risk of bias), and at review-level (e.g., incomplete retrieval of identified research, reporting bias).	n/a
Conclusions	26	Provide a general interpretation of the results in the context of other evidence, and implications for future research.	28
		**FUNDING**	
Funding	27	Describe sources of funding for the systematic review and other support (e.g., supply of data); role of funders for the systematic review.	n/a

ELECTRONIC SUPPLEMENTARY MATERIAL APPENDIX S2 (SEARCH STRATEGY)

TABLE S3.

Database	retrieval strategy
**WOS**	(TS = (“remote ischemic preconditioning” OR “remote ischaemic preconditioning” OR “remote preconditioning” OR “remote conditioning” OR “remote ischemic conditioning” OR “remote ischaemic conditioning” OR “transient limb ischemia” OR “muscle ischemia” OR “ischemic preconditioning”)<br>AND<br>TS = (“performance” OR sport* OR exercise OR “strength training” OR running OR swimming OR cycling OR athletes OR “athletic performance”))
**Pubmed**	((“remote ischemic preconditioning” [Title/Abstract] OR “remote ischaemic preconditioning” [Title/Abstract] OR “remote preconditioning” [Title/Abstract] OR “remote conditioning” [Title/Abstract] OR “remote ischemic conditioning” [Title/Abstract] OR “remote ischaemic conditioning” [Title/Abstract] OR “transient limb ischemia” [Title/Abstract] OR “muscle ischemia” [Title/Abstract] OR “ischemic preconditioning” [Title/Abstract])<br>AND<br>(“performance” [Title/Abstract] OR “sport*” [Title/Abstract] OR “exercise” [Title/Abstract] OR “strength training” [Title/Abstract] OR “running” [Title/Abstract] OR “swimming” [Title/Abstract] OR “cycling” [Title/Abstract] OR “athletes” [Title/Abstract] OR “athletic performance” [Title/Abstract]))
**Embase**	(‘remote ischemic preconditioning’:ti,ab OR ‘remote ischaemic preconditioning’:ti,ab OR ‘remote preconditioning’:ti,ab OR ‘remote conditioning’:ti,ab OR ‘remote ischemic conditioning’:ti,ab OR ‘remote ischaemic conditioning’:ti,ab OR ‘transient limb ischemia’:ti,ab OR ‘muscle ischemia’:ti,ab OR ‘ischemic preconditioning’:ti,ab)<br>AND<br>(‘performance’:ti,ab OR ‘sport*’:ti,ab OR ‘exercise’:ti,ab OR ‘strength training’:ti,ab OR ‘running’:ti,ab OR ‘swimming’:ti,ab OR ‘cycling’:ti,ab OR ‘athletes’:ti,ab OR ‘athletic performance’:ti,ab

ELECTRONIC SUPPLEMENTARY MATERIAL APPENDIX S3 (CHARACTERISTICS OF THE INCLUDED STUDIES)

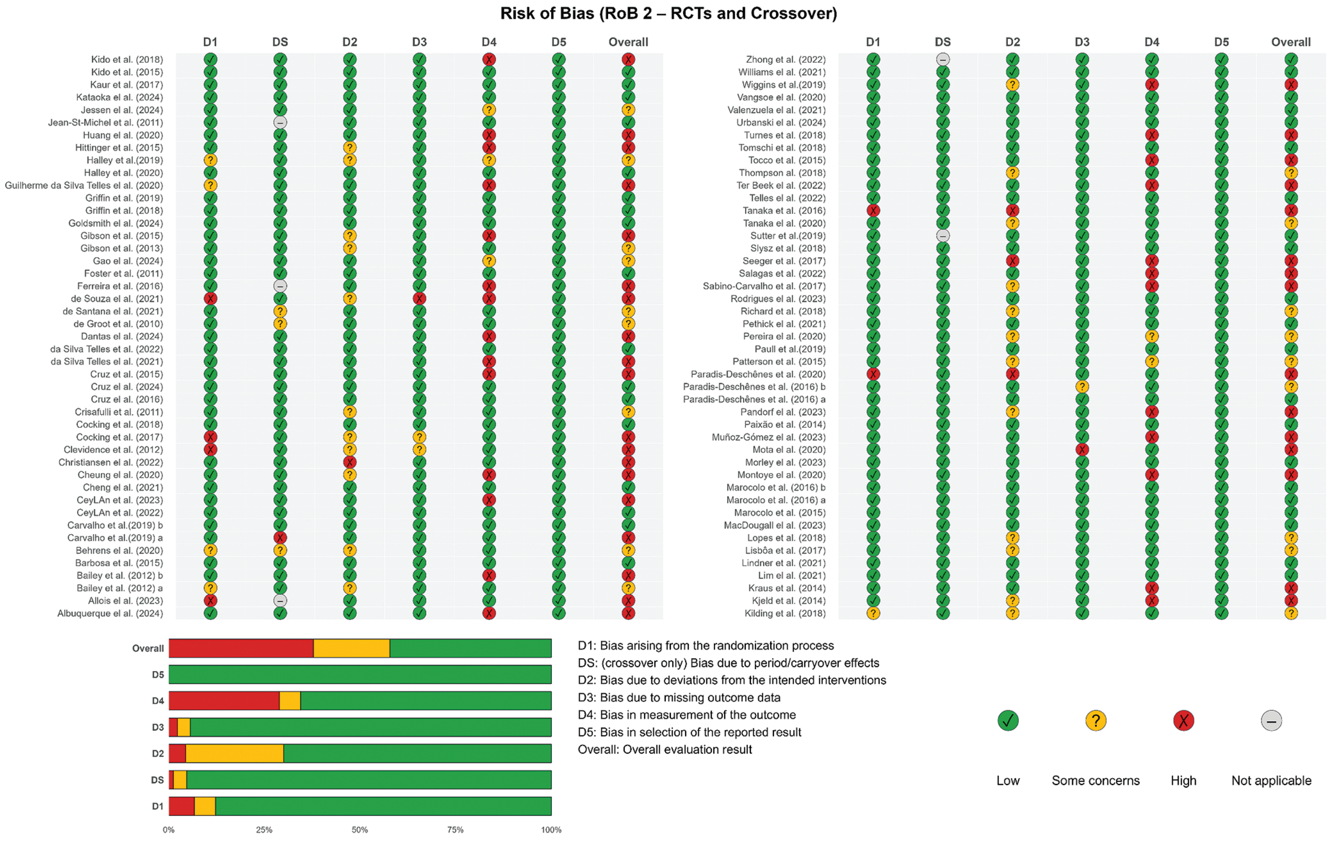

FIG. S2. Physical performance risk of bias assessment

ELECTRONIC SUPPLEMENTARY MATERIAL APPENDIX S4 (CHARACTERISTICS OF THE INCLUDED STUDIES)

TABLE S4. Characteristics of the included studies

Study	Study design	Compa-rison	Participant population	Age	Experence level	IPC sets	Pressure (mmHg)	IPC limb	Interval (h)	Timing	WU level	Type of exercise	Exercies protocols	performance outcome measure
Albuquerque et al. (2024)	RCOs	IPC *vs.* SHAM	14 black belt taekwondo athletes (M: 8, FM: 6)	18	4	4 × 5 min	187.5	Thighs (bilateral)	0	pre-WU	submax	Taekwondo	1 min low-intensity taekwondo kicking drills, then 3 CMJ (15 s rest).	Jump; TE
Allois et al. (2023)	RCTs	IPC *vs.* SHAM	19 healthy participants (M: 15; FM: 4)	27.5	1	3 × 5 min	250	Thighs (bilateral)	0.5	pre-WU	submax	RT	Biceps curl contraction test: 3 s contractions at 80% MVC (3 s rest) until failure.	Strength; RM
Bailey et al. (2012) (a)	RCOs	IPC *vs.* SHAM	13 healthy males at medium training level	25	2	4 × 5 min	220	Thighs (bilateral)	0.33 and 1	pre-WU	submax	Running	Graded maximal running test: 5 × 3 min followed by 2-min stages until volitional exhaustion; 5 km TT after 45 min rest.	TC; $\dot{V}O_2$
Bailey et al. (2012) (b)	RCOs	IPC *vs.* SHAM	13 healthy males at medium training level	25	2	4 × 5 min	220	Thighs (bilateral)	0 and 0.75	pre-WU	submax	Running	Graded maximal running test: 5 × 3 min (30 s rest); 5 km TT after 45 min rest.	TC; $\dot{V}O_2$

TABLE S4. Continue.

Study	Study design	Compa-rison	Participant population	Age	Experence level	IPC sets	Pressure (mmHg)	IPC limb	Interval (h)	Timing	WU level	Type of exercise	Eexrcies protocols	performance outcome measure
Barbosa et al. (2015)	RCOs	IPC *vs.* SHAM	22 healthy males	25	1	3 × 5 min	200	Thighs (bilateral)	0.42	pre-test	NO	RT	MVC Test: 60 handgrip reps at 45% MVC until failure.	RM
Behrens et al. (2020)	RCOs	IPC *vs.* SHAM	16 healthy adult males	26	2	3 × 5 min	254	Thighs (bilateral)	0.33	pre-WU	submax	RT	2 × 5 s at 50%, 70%, and 90% MVC; unilateral isometric knee extension until failure at 20% MVC.	TE; Strength
Carvalho et al. (2019) (a)	RCOs	IPC *vs.* SHAM	10 resistance--trained males	22	2	4 × 5 min	250	Rlternate thighs (bilateral)	0.5	pre-WU	max	RT	1 × 85% 1RM MVC until concentric failure.	RM
Carvalho et al.(2020)	RCOs	IPC *vs.* SHAM	10 resistance--trained males	22	2	4 × 5 min	250	Rlternate thighs (bilateral)	0.5	pre-WU	max	RT	3 × 3 s MVC (2 min rest);	Strength
CeyLAn et al. (2022)	RCOs	IPC *vs.* SHAM	10 elite male judo athletes	20	5	3 × 5 min	220	Rlternate thighs (bilateral)	NA	pre-test	NO	Judo	15 s, 30 s, and 30 s test intervals to throw a similarly-sized partner as fast as possible using "ippon-seoi-nage" technique (10 s rest);	RM
CeyLAn et al. (2023)	RCOs	IPC *vs.* SHAM	13 elite male judo athletes	18.6	5	3 × 5 min	180	Rlternate thigh (unilateral)	1	pre-WU	submax	Judo	Judo Turn Task immediately followed by IPC; record turn metrics with no start or end, plus 30 min and 60 min related markers.	CMJ; strength
Cheng et al. (2021)	RCOs	IPC *vs.* CON	15 male college basketbPhysical performance players	21	3	4 × 5 min	220	Thighs (bilateral)	0.25	pre-WU	submax	Running	6 × 30 s Wingate-style sprints (4 min rest);	P_{avg}; P_{peak}
Cheung et al. (2020)	RCOs	IPC *vs.* SHAM/CON	16 adults engaged in recreational activities(M: 8, FM: 8)	21	1	4 × 5 min	191	Rlternate thigh (unilateral)	NA	pre-WU	submax	Cycling	Incremental exercise to exhaustion test: male participants start at 100 W, female participants at 75 W, increasing by 1 W every 3-4 s.	TC; $\dot{V}O_2$
Christiansen et al. (2022)	RCOs	IPC *vs.* SHAM	12 healthy males with physicPhysical performancey active	28	1	4 × 5 min	220	Thighs (bilateral)	0.18	pre-WU	submax	Cycling	12 min rest after warm up, 4 min cycling test (simulated 4,000 metre individual pursuit).	$\dot{V}O_2$; P_{avg}
Clevidence et al. (2012)	RCOs	IPC *vs.* CON	12 male cyclists	26.7	3	3 × 5 min	220	Rlternate thigh (unilateral)	0.083	pre-test	NA	Cycling	P_{peak} cycling at 30%, 50%, and 70% of P_{peak} for 5 min each, then cycling at 90% of P_{peak} until exhaustion.	$\dot{V}O_2$
Cocking et al. (2017)	RCOs	IPC *vs.* SHAM	14 amateur cyclists	29	3	4 × 5 min	220	Alternate arms and thighs (both sides)	NA	pre-test	NA	Cycling	1 h Timed Cycling Test.	P_{avg}

TABLE S4. Continue.

Study	Study design	Compa-rison	Participant population	Age	Experence level	IPC sets	Pressure (mmHg)	IPC limb	Interval (h)	Timing	WU level	Type of exercise	Eexrcies protocols	performance outcome measure
Cocking et al. (2018)	RCOs	IPC *vs.* SHAM	12 male cyclists	36	3	4 × 5 min	220	Thighs (bilateral)	0.33	pre-WU	submax	Cycling	375 kJ TT immediately after the warm up.	TC; $\dot{V}O_2$; P_{avg}
Crisafulli et al. (2011)	RCOs	IPC *vs.* SHAM/CON	17 healthy males	35.2	1	3 × 5 min	50 > SBP	Thighs (bilateral)	0.083	pre-WU	submax	Cycling	Incremental exercise to exhaustion test: Start at 25 W, increase by 25 W/min until exhaustion. Supramaximal test at 130% P_{peak}.	TC; $\dot{V}O_2$; P_{avg}
Cruz et al. (2016)	RCOs	IPC *vs.* SHAM	15 amateur male cyclists	28	1	4 × 5 min	220	Thighs (bilateral)	0.5	pre-WU	submax	Running	After 5 min rest. 60 s seated sprint with 7.5% body weight resistance.	MAOD; $\dot{V}O_2$; P_{peak}
Cruz et al. (2024)	RCOs	IPC *vs.* SHAM	10 healthy adult males	27	0	4 × 5 min	220	Thighs (bilateral)	0.75	pre-test	NO	RT	2 min intervals with 10 MVC, each lasting ~1.4 s.	Strength
Cruz et al. (2015)	RCOs	IPC *vs.* SHAM	12 amateur cyclists	28	3	4 × 5 min	220	Thighs (bilateral)	1.5	pre-WU	submax	Cycling	After 5 min rest. Incremental exercise to exhaustion test: increase to 100% P_{peak}, until exhaustion or rpm drop > 5 for 5 s.	$\dot{V}O_2$
da Silva Telles et al. (2021)	RCOs	IPC *vs.* SHAM	16 healthy adults (M: 18, FM: 6)	24.8	2	4 × 5 min	220	Alternate arms (bilateral)	0.083	pre-WU	submax	RT	45° leg press RM test: 80% 1 RM	RM
da Silva Telles et al. (2022)	RCOs	IPC *vs.* SHAM/CON	16 active elderly women	68.1	1	3 × 5 min	132	Alternate arms (bilateral)	0.083	pre-warm-up	submax	Walking	6-min walk test	TC
Dantas et al. (2024)	RCOs	IPC *vs.* SHAM	21 trained males	21	1	3 × 5 min	220	Thighs (bilateral)	0	pre-WU	submax	Running	6 × 35 m maximal sprints (10 s rest).	TC; P_{avg}; P_{peak}
de Groot et al. (2010)	RCOs	IPC *vs.* CON	15 healthy adults (M: 12, FM: 3)	27	3	3 × 5 min	220	Thighs (bilateral)	0.083	pre-test	NA	Cycling	Incremental exercise to exhaustion test: start at 50 W, increase by 50 W every 4 min, then by 150 W every 4 min, until exhaustion.	$\dot{V}O_2$; P_{peak}
de Santana et al. (2021)	RCOs	IPC *vs.* SHAM	24 healthy adults (M: 18, FM: 6)	25.8	1	3 × 5 min	220	Rlternate thighs (bilateral)	4	pre-WU	submax	RT	45° leg press RM test: 50% RM, repeated with random 72-h intervals.	RM
de Souza et al. (2021)	RCOs	IPC *vs.* SHAM	9 healthy young males	22.4	1	3 × 5 min	SBP < 50	Thighs (bilateral)	0.13	pre-WU	submax	RT	Unilateral MVIC (10 s), rest interval of 3 min and 3 sets with 2-min rest of dynamicunilateral leg extension (75% 1RM)	RM; Strength

TABLE S4. Continue.

Study	Study design	Compa-rison	Participant population	Age	Experence level	IPC sets	Pressure (mmHg)	IPC limb	Interval (h)	Timing	WU level	Type of exercise	Eexrcies protocols	performance outcome measure
Ferreira et al. (2016)	RCTs	IPC *vs.* SHAM	23 college swimmers	23.9	2	3 × 5 min	220	Thighs (bilateral)	0.17	pre-WU	submax	Swimming	Rest for 5 min after warm-up.6 × 50 m sprints (20 s rest), Borg scale 5–6, effort level 2–3.	TC
Foster et al. (2011)	RCOs	IPC *vs.* CON	8 experienced cyclists (M: 6, FM: 2)	39	3	4 × 5 min	SBP < 20	Thigh (unilateral)	1.5	post-warm-up and pre-test	submax	Cycling	4–5 km TT	TC
Gao et al. (2024)	RCOs	IPC *vs.* SHAM/CON	22 healthy males	26.8	1	3 × 5 min	220	Arms (bilateral)	0.5	pre-WU	submax	Cycling	5 min rest after warm-up.5 sets of 10 s (40 s rest) maximal sprints;	P_{avg}; P_{peak}
Gibson et al. (2013)	RCOs	IPC *vs.* SHAM/CON	25 healthy adults (M: 16, FM: 9)	22.9	2	3 × 5 min	220	Rlternate thighs (bilateral)	0.25	pre-WU	submax	Running	submaximal 30 m sprints × 2; 3 times max sprints of 10, 20, and 30 m (1 min rest);	TC
Gibson et al. (2015)	RCOs	IPC *vs.* SHAM/CON	16 well-trained team athletes (M: 7, FM: 9)	24	2	3 × 5 min	220	Rlternate thigh (unilateral)	0.083	pre-WU	submax	Running	2 × 3 sprints followed by 5 × 6 s max effort sprints with 7.5% body weight resistance.	P_{peak}
Goldsmith et al. (2024)	RCOs	IPC *vs.* SHAM/CON	10 recreationPhysical performancey active participants (M: 5, FM: 5)	31.8	1	4 × 5 min	220	Thighs (bilateral)	0.16	pre-test	NA	Cycling	Incremental exercise to exhaustion test: (10 W/min) until exhaustion.	$\dot{V}O_2$; P_{peak}
Griffin et al. (2018)	RCOs	IPC *vs.* SHAM	12 amateur male athletes	30	3	4 × 5 min	220	Thighs (bilateral)		pre-WU	submax	Cycling	3 min high-intensity cycling at 110 rpm and 10 min recovery cycling.	$\dot{V}O_2$; P_{peak}
Griffin et al. (2019)	RCOs	IPC *vs.* SHAM	12 healthy males engaged in recreational team sports	22	3	4 × 5 min	220	Rlternate thigh (bilateral)	0.25	pre-WU	submax	Running	Repeated sprints protocol: 3 × 6 shuttle sprints (15 m each), alternating between standing and seated recovery, with passive recovery between sets.	TC
Guilherme da Silva Telles et al. (2020)	RCOs	IPC *vs.* SHAM	16 healthy adult males	24.8	2	4 × 5 min	220	Thigh (unilateral)	0.75	pre-WU	submax	RT	3 sets of training at 80% 1RM until concentric failure.	RM
Halley et al. (2020)	RCOs	IPC *vs.* CON	8 well-trained male canoe athletes	21	4	4 × 5 min	220	Thighs (bilateral)	0.33 aad 0.66	pre-WU	submax	rowing	1000 m rowing TT.	TC; $\dot{V}O_2$; P_{avg}
Halley et al. (2019)	RCOs	IPC *vs.* SHAM	11 resistance-trained males	23.1	1	3 × 5 min	220	Thighs (bilateral)	0.16	pre-WU	submax	RT	3 × 5 min (120°/s extension; 300°/s flexion) at 40 cycles per min.	Strength

TABLE S4. Continue.

Study	Study design	Compa-rison	Participant population	Age	Experence level	IPC sets	Pressure (mmHg)	IPC limb	Interval (h)	Timing	WU level	Type of exercise	Eexrcies protocols	performance outcome measure
Hittinger et al. (2015)	RCOs	IPC *vs.* CON	15 male cyclists and triathletes	29.9	2	4 × 5 min	SBP < 10–20	Thighs (bilateral)	0.75	pre-test	NA	Cycling	(normoxic and hypoxic): 10 min submaximal exercise at 55% W_{peak}, increasing 30 W every 2 min until exhaustion.	P_{peak}
Huang et al. (2020)	RCOs	IPC *vs.* SHAM	14 healthy non-athletic males	26	1	3 × 5 min	SBP < 50	Thighs (bilateral)	0.083	pre-WU	submax	RT	3 × maximal isokinetic knee extensions at 30, 150, and 270 deg/s; 30 × isokinetic endurance reps at 180 deg/s.	Strength; P_{avg}
Jean-St-Michel et al. (2011)	RCTs	IPC *vs.* SHAM	16 elite swimmers(M: 8, FM: 8)	18.8	4	4 × 5 min	SBP < 15	Arm (unilateral)	0	pre-WU	submax	Swimming	100 m swimming	TC
Jessen et al. (2024)	RCOs	IPC *vs.* SHAM	11 healthy, well-trained young males	25	2	3 × 2 min	180	Thighs (bilateral)	0.16	pre-WU	sumax	Cycling	4 min TT;	P_{avg}
Kataoka et al. (2024)	RCOs	IPC *vs.* SHAM/CON	39 healthy adults	26.5	1	4 × 5 min	110-150% AOP	Arm (unilateral)	0.05	pre-test	NA	RT	2 × 75 s maximal isokinetic unilateral elbow flexion/ extension (2 min rest). Intervals between tests: 3-10 days.	Strength; P_{avg}
Kaur et al. (2017)	RCOs	IPC *vs.* SHAM	18 runners (M: 12, FM: 6)	27	1	3 × 5 min	220	Thighs (bilateral)	0.25	pre-WU	sumax	Running	incremental submaximal (65–85% $\dot{V}O_{2max}$) treadmill running protocols 3 × 5 min	$\dot{V}O_2$
Kido et al. (2015)	RCOs	IPC *vs.* CON	15 healthy active males	24	1	3 × 5 min	> 300	Thighs (bilateral)	0.083	pre-test	NA	Cycling	Work-to-Work Test: gradual increase in intensity every 3 min by 30 W, reaching 90% GET and 90% of max.	TE; $\dot{V}O_2$
Kido et al. (2018)	RCOs	IPC *vs.* CON	13 healthy active males	22.5	1	3 × 5 min	250	Thighs (bilateral)	0.083	pre-test	NA	Cycling	Work-to-Work Test: low intensity: 30 W for 3 min; medium intensity: 90% GET for 4 min; high intensity: 70% $\dot{V}O_{2max}$ until exhaustion.	TE; $\dot{V}O_2$
Kilding et al. (2018)	RCOs	IPC *vs.* SHAM	8 healthy, well-trained male cyclists	27	3	4 × 5 min	200	Thighs (bilateral)	0.083	pre-WU	submax	Cycling	Incremental and square-wave exercise tests;4 km cycling TT.	TC; $\dot{V}O_2$
Kjeld et al. (2014)	RCOs	IPC *vs.* CON	14 oarsmen (M: 10, FM: 4)	28	4	4 × 5 min	SBP < 40	Forearm (unilateral)	0.5	post-warm-up and pre-test	submax	rowing	1000 m rowing TT.	TC

TABLE S4. Continue.

Study	Study design	Compa-rison	Participant population	Age	Experence level	IPC sets	Pressure (mmHg)	IPC limb	Interval (h)	Timing	WU level	Type of exercise	Eexrcies protocols	performance outcome measure
Kraus et al. (2014)	RCOs	IPC *vs.* SHAM	43 young adults in leisure activities (M: 27, FM: 16)	22.7	1	4 × 5 min	NA	Arm (unilateral)	0.25	pre-test	NA	Cycling	4 × 30 s Wingate anaerobic test, 150 rpm, 9% body weight load, 2 min rest intervals.	P_{avg}; P_{peak}
Lim et al. (2021)	RCOs	IPC *vs.* SHAM	8 Singapore national badminton players (M: 6, FM: 2)	23.1	4	4 × 5 min	250	Arm (bilateral)		pre-WU	submax	Badminton	50 min badminton specific program, with 30 min rest followed by CMJ, sprint test and Yo-Yo intermittent recovery test.	TC; Jump; P_{avg}; P_{peak}
Lindner et al. (2021)	RCOs	IPC *vs.* SHAM	14 NCAA Division II athletes (M: 12, FM: 2)	20	3	3 × 5 min	100% AOP	Thighs (bilateral)	0	pre-WU	submax	Running; Jump	25 min performance test (vertical jump, reactive strength index, standing broad jump, 10 m sprint, agility).	TC; Jump
Lisbôa et al. (2017)	RCOs	IPC *vs.* SHAM	11 male competitive swimmers	20	3	4 × 5 min	220	Thighs (bilateral),Arm (unilateral)	1, 2 and 8	pre-WU	submax	Swimming	50 m freestyle TT: 3 consutive tests.	TC
Lopes et al. (2018)	RCOs	IPC *vs.* SHAM	15 healthy males	25	1	3 × 5 min	220	Thighs (bilateral)	0.17	pre-WU	max	Running	10 rest after warm-up.3 sets of 6 × 15 m shuttle sprints (180° direction change), 20 s active recovery.	TC; $\dot{V}O_2$
MacDougall et al. (2023)	RCOs	IPC *vs.* CON	12 well-trained rock climbers (M: 9, FM: 3)	30.7	2	5 × 2 min	> 300	Arm (unilateral)	0.33	post-warm-up and pre-test	submax	Rock climbing	Constant-intensity climbing hangboard test until failure.	TE; Strength
Marocolo et al. (2015)	RCOs	IPC *vs.* SHAM/CON	15 amateur swimmers	21	2	4 × 5 min	220	Rlternate arm (unilateral)	0.083	pre-WU	submax	Swimming	100 m freestyle test.	TC
Marocolo et al. (2016) (a)	RCOs	IPC *vs.* SHAM/CON	13 healthy males	25.9	2	4 × 5 min	220	Alternate thigh (unilateral)	0.083	pre-WU	submax	RT	3 × maximum leg extension (2 min rest) with the predetermined 12 RM load	RM
Marocolo et al. (2016) (b)	RCOs	IPC *vs.* SHAM	21 healthy males	27.3	2	4 × 5 min	220	Rlternate arm and thigh (unilateral)	0.07	pre-WU	submax	RT	Resistance Training Test: 12 RM load bicep curl (20 reps), followed by 3 × leg extensions (60% 12 RM, 2 min rest);	RM
Montoye et al. (2020)	RCOs	IPC *vs.* SHAM	12 healthy adults (M: 5, FM: 7)	20.4	1	3 × 5 min	220	Thigh (unilateral)	0	pre-WU	submax	Running	2.4 km test	TC
Morley et al. (2023)	RCOs	IPC *vs.* CON	15 healthy and recreationPhysical performancey active adults (M: 9, FM: 6)	27	1	4 × 5 min	stop arterial inflow	Thighs (bilateral)	0.083	pre-WU	submax	Cycling	4 km test	TC; P_{avg}
Mota et al. (2020)	RCOs	IPC *vs.* SHAM	20 healthy adult females	23.1	1	3 × 3 min + 2 min reperfusion	SBP < 50	Arms (bilateral)	0.1	pre-WU	submax	Arm crank exercise	$speed_{max}$ 3 min arm cycling exercise.	P_{avg}; P_{peak}

TABLE S4. Continue.

Study	Study design	Compa-rison	Participant population	Age	Experence level	IPC sets	Pressure (mmHg)	IPC limb	Interval (h)	Timing	WU level	Type of exercise	Eexrcies protocols	performance outcome measure
Muñoz-Gómez et al. (2023)	RCOs	IPC *vs.* SHAM	9 older adults	69.1	0	3 × 5 min	SBP < 50	Thighs (bilateral)	0.083	pre-test	NA	Walking	20 min walking at 65-75% HR_{max}, RPE scale 12-13.	TC; Strength
Paixão et al. (2014)	RCOs	IPC *vs.* SHAM	15 amateur cyclists	30.2	2	4 × 5 min	250	Rlternate thigh (unilateral)	0.2	pre-WU	submax	Cycling	2 × Wingate Test: After each test, 30 s sprints at 0.10 kg/kg load (10 min rest).	P_{peak}
Pandorf et al. (2023)	RCOs	IPC *vs.* SHAM	16 university swimmers (M: 5, FM: 11)	22	3	3 × 5 min	40–220	Thighs (bilateral)	0.2	pre-WU	submax	Swimming	swimming TT (250 m/500 m).	TC
Paradis-Deschênes et al. (2016) (a)	RCOs	IPC *vs.* SHAM	10 strength-trained males	25	2	3 × 5 min	200	Right thigh (unilateral)	0.3	pre-WU	submax	RT	5 × 5 s maximal knee extension test (1 min rest).	Strength
Paradis-Deschênes et al. (2016) (b)	RCOs	IPC *vs.* SHAM	17 strength-trained adults (M: 9, FM: 8)	25	2	3 × 5 min	200	Right thigh (unilateral)	0.3	pre-WU	submax	RT	5 × 5 s maximal knee extension test (1 min rest).	Strength
Paradis-Deschênes et al. (2020)	RCOs	IPC *vs.* CON	9 well-trained male road cyclists, runners, and triathletes	16.4	3	3 × 5 min	220	Rlternate thigh (unilateral)	0.43	pre-WU	submax	Cycling	2 sets 5 km TT;	TC
Patterson et al. (2015)	RCOs	IPC *vs.* SHAM	14 healthy males	22.9	0	4 × 5 min	220	Thighs (bilateral)	0.5	pre-WU	submax	Running	Repeated Sprints: 12 × 6 s sprints, 1.0 Nm/kg torque load.	$\dot{V}O_2$; P_{peak}; P_{avg}
Paull et al. (2019)	RCOs	IPC *vs.* SHAM	10 NCAA Division I middle- and long-distance runners (M: 6, FM: 4)	21	4	4 × 5 min	220	Arm (right side)	0.75	pre-WU	submax	Running	Running at 110% $\dot{V}O_{2max}$ (~20.3 km/h, 5% incline) for > 2 min to exhaustion.	TE; $\dot{V}O_2$
Pereira et al. (2020)	RCOs	IPC *vs.* SHAM/CON	20 healthy adults (M: 10, FM: 10)	28.5	0	3 × 5 min	225	Non-dominant leg thighs and upper arms	0	pre-test	NA	RT	MVC test lasting 3-4 s, performed at least twice (120 s rest).	TE
Pethick et al. (2021)	RCOs	IPC *vs.* SHAM	10 healthy adults (M: 6, FM: 4)	25.9	0	3 × 5 min	225	Thigh (unilateral)	0.33	pre-test	NA	RT	40% MVC intermittent isometric knee extension contractions (6 s, rest 4 s) to task failure	TE
Richard et al. (2018)	RCOs	IPC *vs.* SHAM	9 elite long track speed skaters (M: 7, FM: 2)	23.3	4	3 × 5 min	SBP < 30	Arms (bilateral)	1	pre-WU	submax	Skating	20-40 min individual race warm-up, followed by 1000 m ice TT.	TC
Rodrigues et al. (2023)	RCOs	IPC *vs.* SHAM/CON	15 resistance-trained males	29.9	2	3 × 5 min	170	Arms (unilateral))	0.17	pre-WU	submax	RT	1 RM test	Strength

TABLE S4. Continue.

Study	Study design	Compa-rison	Participant population	Age	Experence level	IPC sets	Pressure (mmHg)	IPC limb	Interval (h)	Timing	WU level	Type of exercise	Eexrcies protocols	performance outcome measure
Sabino-Carvalho et al. (2017)	RCOs	IPC *vs.* SHAM/CON	18 healthy adults (M: 14, FM: 4)	23.15	1	4 × 5 min	220	Rlternate thigh (unilateral)	0.16	pre-test	NA	Running	8 km/h, +1 km/h/ min to exhaustion. 6 min baseline, +1 km/h/stage to exhaustion. 2 min at 60% Vpeak, +0.5 km/h to exhaustion.	TE; $\dot{V}O_2$
Salagas et al. (2022)	RCOs	IPC *vs.* CON	12 healthy males	25.8	1	1 × 5 min	100% AOP	Arms (bilateral)	0	post-warm-up and pre-test	max	RT	perform 4 × bench press at 60% of 1RM (each lasting 12 s,12 s rest) .	RM
Seeger et al. (2017)	RCOs	IPC *vs.* SHAM	12 healthy adults (M: 10, FM: 2)	31	2	4 × 5 min	220	Thighs (bilateral)	0 and 24	pre-WU	submax	Running	5 km TT.	TC
Slysz et al. (2018)	RCOs	IPC *vs.* CON	12 healthy males	22	1	3 × 5 min	220	Thighs (bilateral)	0.17	pre-WU	submax	Walking	30-second Wingate test and subsequent (after 25 minutes of rest) incremental maximal aerobic test	P_{peak}
Sutter et al. (2019)	RCTs	IPC *vs.* SHAM	69 healthy older adults	61.1	0	5 × 5 min	20 > SBP	Arms (bilateral)	0	pre-test	NA	Balance	Standing on a platform with feet facing forward, maintaining balance for as long as possible, each trial lasting 30 s.	Balance
Tanaka et al. (2020)	RCOs	IPC *vs.* CON	14 healthy adult males	22.1	0	3 × 5 min	300	Thigh (unilateral)	0.083	pre-test	NA	RT	20% MVC sustained isometric unilateral knee extension until failure.	TE
Tanaka et al. (2016)	RCOs	IPC *vs.* CON	12 healthy males	22	0	3 × 5 min	> 300	Thigh (unilateral)	0.083	pre-test	NA	RT	MVC Test: gradual torque increase to max, 3 × 3 s (1 min rest); Submaximal Fatigue Test: 20% MVC torque until failure.	Strength; TE
Telles et al. (2022)	RCOs	IPC *vs.* SHAM/CON	16 males with recreational training	27.8	1	4 × 5 min	220	Arms (bilateral)	0.17	pre-WU	submax	RT	3 × 3-5 reps at 60-80% perceived max (1 min rest) before strength testing.	Strength
Ter Beek et al. (2022)	RCOs	IPC *vs.* SHAM	14 healthy adult males	24.9	2	4 × 5 min	250	Thighs (bilateral)		pre-WU	submax	Cycling	Incremental exhaustion test: 105 W for 7 min, increasing by 35 W every 2 min at 70 rpm until exhaustion.	$\dot{V}O_2$; P_{peak}
Thompson al. (2018)	RCOs	IPC *vs.* SHAM/CON	18 top Canadian university track and field sprinters (M: 10, FM: 8)	20.85	4	3 × 5 min	220	Thighs (bilateral)	0.25	post-warm-up and pre-test	submax	Running	4 × 20 m sprints (5 min rest).	TC

TABLE S4. Continue.

Study	Study design	Compa-rison	Participant population	Age	Experence level	IPC sets	Pressure (mmHg)	IPC limb	Interval (h)	Timing	WU level	Type of exercise	Eexrcies protocols	performance outcome measure
Tocco et al. (2015)	RCOs	IPC *vs.* SHAM	16 male skilled runners	34.6	3	3 × 5 min	220	Rlternate thigh (unilateral)	0.25	pre-WU	submax	Running	5 km selfpaced run	$\dot{V}0_2$
Tomschi et al. (2018)	RCOs	IPC *vs.* SHAM	10 healthy males	24.3	0	4 × 5 min	120, 220	Right arm (unilateral)	0.083	pre-test	NA	Cycling	incremental power test at 60 rpm until exhaustion.	$\dot{V}0_2$; P_{avg}
Turnes et al. (2018)	RCOs	IPC *vs.* CON	16 male rowers at national and regional level	24	4	3 × 5, 3 × 10 min	220	Thigh (unilateral)	0.5	pre-WU	submax	Rowing	5 min rest after warm-up. 2000 m Rowing TT.	TC; $\dot{V}0_2$; P_{avg}
Urbanski et al. (2024)	RCOs	IPC *vs.* SHAM	43 physicPhysical performancey active individuals (M: 24, FM: 19)	20	2	1 × 5 min	220	Upper and lower limbs simultaneously (unilateral)	0.083	post-warm-up and pre-test	submax	Rowing	500 m Physical performance-out rowing trial.	TC; P_{peak}; P_{avg}
Valenzuela et al. (2021)	RCOs	IPC *vs.* SHAM	16 male healthy adults	23	1	3 × 5 min	220	Arms (bilateral)	0.5	pre-WU	submax	RT	resistance exercise: 3 × 3 reps to failure at 60% 1RM, 2 min passive rest between sets.	RM; Strength
Vangsoe et al. (2020)	RCOs	IPC *vs.* CON	12 male well-trained cyclists	32	2	3 × 5 min	200	Thighs (bilateral)	0.05	pre-WU	submax	Cycling	1 km test.	TC; P_{peak}; P_{avg}
Wiggins et al. (2019)	RCOs	IPC *vs.* SHAM	13 male athletes regularly engaging in endurance training	24	1	4 × 5 min	220	Thighs (bilateral)	0.17	pre-WU	submax	Cycling	6 min cycling at intensities 15% below anaerobic threshold and 85% $\dot{V}0_{2max}$, then 5 km TT.	TC; $\dot{V}0_2$
Williams et al. (2021)	RCOs	IPC *vs.* SHAM	20 international and national level swimmers (M: 14, FM: 6)	20	5	4 × 5 min	194	Thighs (bilateral)	2 and 24	pre-WU	submax	Swimming	100 m and 200 m TT.	TC
Zhong et al. (2022)	RCTs	IPC *vs.* SHAM	79 healthy adult males	23.96	0	4 × 5 min	180	Arms (bilateral)	0.5	pre-WU	submax	Cycling	Phase 1: 4 km altitude simulation with 0.75 h hypoxic treatment, 4.25 h graded exercise test ($\dot{V}0_{2max}$), 0.5 h recovery; Phase 2: 8 days post-IPC with direct physiological variable testing.	$\dot{V}0_2$

IPC: ischemic preconditioning; SHAM: sham intervention; CON: no intervention; *vs.*: versus; sets:Ischaemia and reperfusion duration for IPC; WU: warm-up; M: male; FM: female; NA: unknown; NO: no warm-up component; RT: resistance training; TC; Time to complete; TE: Time to exhausted; RM: maximum number of repetitions; TT: Time trial; CMJ: Countermovement Jump; s: second; min: minute; h: hour; P_{avg}: Average Power Output; P_{peak}: Peak Power Output; MVC: Maximum Voluntary Contraction; MIVC; Maximum Isotonic Voluntary Contraction; FSKT: Frequency Speed of Kick Test; m:meter; km: kilometer; W: watt; reps: repetitions; SBP: systolic blood pressure; AOP: arterial occlusion pressure;

ELECTRONIC SUPPLEMENTARY MATERIAL APPENDIX S5 (GOODNESS-OF-FIT AND CORRELATION COEFFICIENT COMPARISON RESULTS)

TABLE S5. Comparative analysis of the goodness of fit for exercise performance with respect to the interval time of warm-up content without differentiation.

PHYSICAL PERFORMANCE (interval)	linear	Cubic Polynomial Model_2	Cubic Polynomial Model_3	Restricted Cubic Spline Model_4	Natural Cubic Spline Model	Restricted Cubic Spline Model_3
logLik:	-110.4756	-109.5485	-109.4249	-109.3402	-109.3402	-109.869
deviance:	220.9512	219.0969	218.8498	218.6804	218.6804	219.7379
AIC:	230.9512	231.0969	232.8498	232.6804	232.6804	231.7379
BIC:	249.3861	253.1984	258.611	258.4416	258.4416	253.8394
AICc:	231.1588	231.3896	233.2428	233.0734	233.0734	232.0306

TABLE S6. Comparative analysis of the goodness of fit between warm-up interval durations and exercise performance

pre-wu (interval)	linear	Cubic Polynomial Model_2	Cubic Polynomial Model_3	Restricted Cubic Spline Model_4	Natural Cubic Spline Model	Restricted Cubic Spline Model_3
logLik:	-37.98233	-37.19718	-37.19718	-36.96561	-36.9654	-37.60517
deviance:	75.96465	74.39437	74.39437	73.93121	73.93079	75.21034
AIC:	85.96465	86.39437	86.39437	87.93121	87.93079	87.21034
BIC:	102.32965	106.00152	106.00152	110.77005	110.76962	106.81749
AICc:	86.28211	86.84357	86.84357	88.53662	88.5362	87.65954

TABLE S7. Comparative analysis of the goodness of fit between intervals without warm-up and exercise performance

pre-test (interval)	linear	Cubic Polynomial Model_2	Cubic Polynomial Model_3	Restricted Cubic Spline Model_4	Natural Cubic Spline Model	Restricted Cubic Spline Model_3
logLik:	-59.00176	-58.77829	-58.22862	-57.57053	-57.56976	-58.50851
deviance:	118.00352	117.55658	116.45724	115.14107	115.13952	117.01701
AIC:	128.00352	129.55658	130.45724	129.14107	129.13952	129.01701
BIC:	139.91366	143.77326	146.9542	145.63803	145.63648	143.2337
AICc:	128.81433	130.72324	132.05724	130.74107	130.73952	130.18368

TABLE S8. Goodness-of-fit analysis of exercise performance across participant age groups

Age (Physical performance)	linear	Cubic Polynomial Model_2	Cubic Polynomial Model_3	Restricted Cubic Spline Model_4	Natural Cubic Spline Model	Restricted Cubic Spline Model_3
logLik:	-109.6076	-108.9525	-108.2293	-105.9413	-109.6076	-109.1073
deviance:	219.2153	217.9051	216.4586	211.8826	219.2153	218.2145
AIC:	229.2153	229.9051	230.4586	225.8826	229.2153	230.2145
BIC:	247.6502	252.0066	256.2198	251.6439	247.6502	252.316
AICc:	229.4229	230.1978	230.8516	226.2756	229.4229	230.5072

TABLE S9. Goodness-of-fit comparison of anaerobic performance across participant age groups

Age (anaerobic)	linear	Cubic Polynomial Model_2	Cubic Polynomial Model_3	Restricted Cubic Spline Model_4	Natural Cubic Spline Model	Restricted Cubic Spline Model_3
logLik:	-65.80637	-65.47321	-64.96391	-63.99098	-65.80637	-64.8716
deviance:	131.61274	130.94643	129.92783	127.98195	131.61274	129.7432
AIC:	141.61274	142.94643	143.92783	141.98195	141.61274	141.7432
BIC:	157.32106	161.76122	165.83712	163.89124	157.32106	160.558
AICc:	141.97637	143.46177	144.62348	142.67761	141.97637	142.2585

TABLE S10. Goodness-of-fit comparison of aerobic performance across participant age groups

Age (aerobic)	linear	Cubic Polynomial Model_2	Cubic Polynomial Model_3	Restricted Cubic Spline Model_4	Natural Cubic Spline Model	Restricted Cubic Spline Model_3
logLik:	-57.74554	-57.17875	-56.49745	-56.36723	-57.74554	-57.21918
deviance:	115.49108	114.35749	112.9949	112.73446	115.49108	114.43837
AIC:	123.49108	124.35749	124.9949	124.73446	123.49108	124.43837
BIC:	135.22898	138.99376	142.51479	142.25434	135.22898	139.07463
AICc:	123.78959	124.81204	125.64106	125.38061	123.78959	124.89291

TABLE S11. Comparison of the goodness of fit of effect sizes for different levels of meta-analyses

Last model results								
PHYSICAL PERFORMANCE(interval)								
	df	AIC	BIC	AICc	logLik	LRT	pval	QE
four-level	4	261.7094	276.6942	261.8392	-126.8547	NA	NA	426.5362
three-level	3	263.8613	275.0999	263.939	-128.9307	4.15198	0.0415859	426.5362
PHYSICAL PERFORMANCE(anaerobic)								
	df	AIC	BIC	AICc	logLik	LRT	pval	QE
four-level	4	132.7224	145.3123	132.9619	-62.36119	NA	NA	242.0826
three-level	3	137.6712	147.1137	137.814	-65.8356	6.948821	0.0083875	242.0826
PHYSICAL PERFORMANCE(aerobic)								
	df	AIC	BIC	AICc	logLik	LRT	pval	QE
three-level	3	123.3172	132.1421	123.4937	-58.65861	NA	NA	180.8781
two-level	2	132.6907	138.574	132.7783	-64.34536	11.37351	0.000745	180.8781

ELECTRONIC SUPPLEMENTARY MATERIAL APPENDIX S6 (STATISTICAL POWER ANALYSIS)

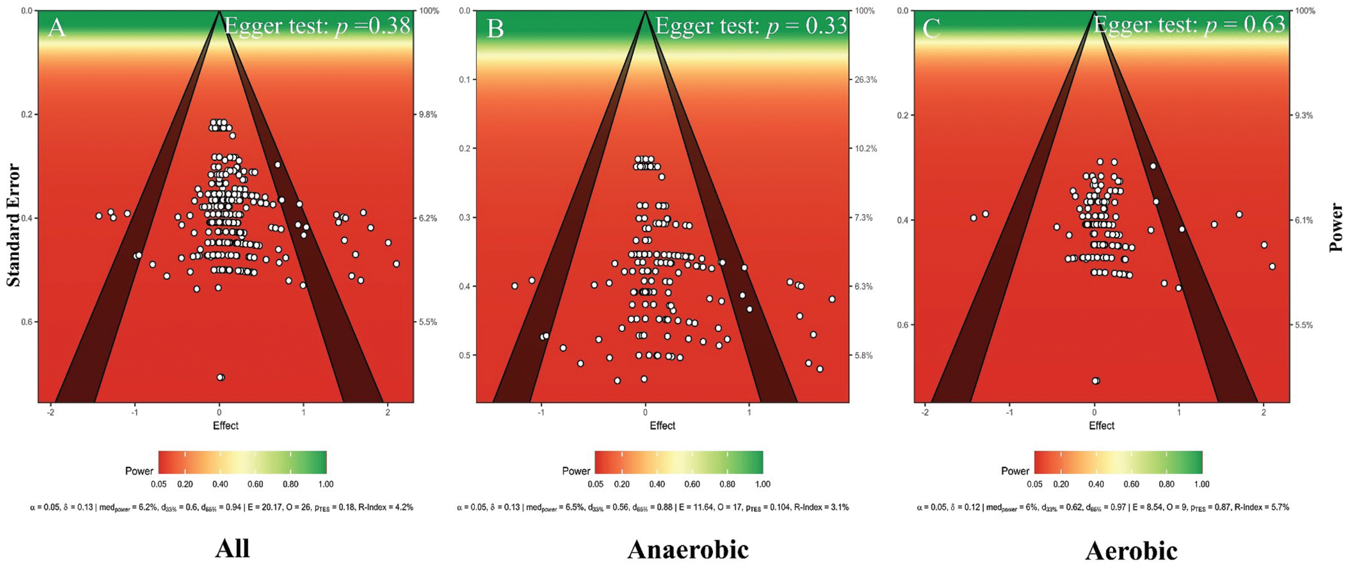

FIG. S3. Statistical power of IPC on athletic performance and Egger's test results
Note: The vertical solid line represents the pooled effect size (Hedges g = -050 and -0.30), the vertical dash line represents the adjusted pooled effect size. Significance contours at .05 and .01 levels are noted by the shaded area. medpower indicates the median power of Physical performance included effect sizes. *d33%* and *d66%* indicate the true effect sizes necessary for achieving 33% and 66% levels of median power. E, O, and PTES show the results of a test of excess significance. R-index denotes the expected replicability of findings.

ELECTRONIC SUPPLEMENTARY MATERIAL APPENDIX S7 (PHYSICAL PERFORMANCE DETAILED RESULTS OF DATA ANALYSIS)

TABLE S12. Effects of IPC on sport performance——Three or Four-level model

Outcome	Studies(K)	Effect size(n)	Effect size	95%CI	95%CI_adj	*p*-value	*p*-value_adj	t-value	*Q* test	I^2-Level 2	I^2-Level 3	I^2-Level 4	PI	*p*-Egger
Physical performance	90	314	0.1318	[0.0571; 0.2066]	[0.0719; 0.1918]	0.0006	< 0.0001	3.4705	426.536	0.00%	9.13%	5.74%	[-0.1787; 0.4424]	0.3854
Anaerobic	49	173	0.1451	[0.063; 0.2271]	[0.0469; 0.2432]	0.0009	0.004	3.5757	242.083	0.00%	13.64%	0.00%	[-0.1429; 0.4331]	0.3295
Aerobic	44	141	0.0994	[-0.0126; 0.2114]	[0.0101; 0.1887]	0.0816	0.0301	2.2517	180.878	0	0.1653	0	[-0.2741; 0.4728]	0.6324

K: number of studies; n:Total number of effects included; 95%CI: Unadjusted 95% confidence interval (range of effect size estimates); 95%CI_adj: Adjusted 95% confidence interval (corrected for multilevel models or heterogeneity);
p-value_adj: Adjusted p-value (e.g., corrected for multiple comparisons or model adjustments); t-value: t-test statistic (for testing significance of the effect size); Q test: Q statistic for heterogeneity test (assessing between-study heterogeneity).
I^2-Level 2/3/4: Heterogeneity proportions in multilevel models; PI: Prediction Interval; *p*-Egger: Egger test p-value to detect publication bias (e.g. p < 0.05 for possible bias);

TABLE S13. Subgroup analyses of IPC on sport performance—Three or Four-level model (1)

Variable	Moderator	Studies (K)	Effect size(n)	Effect size	95%CI	95%CI_adj
Exercise type	aerobic	44	141	0.1254	[0.0169; 0.2339]	[0.0431; 0.2077]
	anaerobic	49	173	0.1368	[0.0414; 0.2322]	[0.0570; 0.2166]
Comparison	IPC *vs.* CON	33	95	0.223	[0.1152; 0.3309]	[0.0942; 0.3519]
	IPC *vs.* SHAM	73	219	0.0961	[0.0162; 0.1759]	[0.0345; 0.1576]
Sex	sexmale	55	182	0.2008	[0.1041; 0.2975]	[0.1192; 0.2824]
	sexmixed	29	97	0.0646	[-0.0569; 0.1862]	[-0.0003; 0.1295]
	sexNA	5	18	-0.112	[-0.4487; 0.2248]	[-0.7221; 0.4982]
	sexfemale	6	17	0.0845	[-0.1206; 0.2895]	[-0.0813; 0.2502]
Experence_level	level2	24	76	0.1845	[0.0409; 0.3282]	[0.0419; 0.3272]
	level0	10	35	0.143	[-0.0992; 0.3852]	[0.0233; 0.2627]
	level1	29	116	0.1508	[0.0280; 0.2736]	[0.0283; 0.2734]
	level3	15	43	0.0819	[-0.1231; 0.2870]	[-0.0100; 0.1739]
	level4	9	37	0.0135	[-0.2370; 0.2640]	[-0.0602; 0.0872]
	level5	3	7	-0.0206	[-0.4335; 0.3922]	[-0.5265; 0.4852]
IPC_sets	4 × 5 min	43	157	0.107	[-0.0004; 0.2144]	[0.0201; 0.1938]
	1 × 5 min	2	7	0.6031	[0.1210; 1.0851]	[-6.6568; 7.8629]
	3 × 2 min	1	1	-0.0166	[-0.9129; 0.8798]	[NA; NA]
	3 × 10 min	1	3	0.1761	[-0.2101; 0.5623]	[-0.0273; 0.3794]
	3 × 3 min + 2 min reperfusion	1	2	-0.0613	[-0.6862; 0.5636]	[-0.0635; -0.0591]
	3 × 5 min	41	138	0.1422	[0.0280; 0.2565]	[0.0516; 0.2329]
	5 × 2 min	1	3	0.1333	[-0.6156; 0.8821]	[-0.7613; 1.0279]
	5 × 5 min	1	1	0.1584	[-0.4117; 0.7284]	[NA; NA]
	8 × 5 min	1	2	0.0191	[-0.4499; 0.4881]	[-0.0985; 0.1367]
Warm-up	NO	22	84	0.0987	[-0.0430; 0.2404]	[-0.0757; 0.2731]
	YES	70	230	0.1433	[0.0577; 0.2289]	[0.0662; 0.2204]

TABLE S13. Subgroup analyses of IPC on sport performance—Three or Four-level model (2)

Variable	p-value	p-value_adj	t-value	F-test	*Q* test	I^2-Level 2	I^2-Level 3	I^2-Level 4	$p_{\text{-diff}}$
Exercise type	0.0237	0.0038	2.2735	$F_{(2,312)} = 6.027$	426.533	0	0.0926	0.0583	0.8707
	0.0051	0.0013	2.8218						
Comparison	0.0001	0.0013	4.0702	$F_{(2,312)} = 8.752$	421.295	0	0.0756	0.0561	0.0221
	0.0185	0.0027	2.3672						
Sex	0.0001	0	4.0857	$F_{(4,310)} = 4.528$	419.73	0	0.0834	0.0613	intrcpt
	0.2963	0.0508	1.0462						0.0804
	0.5135	0.6334	-0.6542						0.08
	0.4183	0.245	0.8105						0.2537
Experence_level	**0.012**	**0.0137**	2.5277	$F_{(6,308)} = 2.37$	424.114	0	0.097	0.0588	no difference
	0.2463	0.0248	1.1616						
	0.0162	0.0181	2.4171						
	0.4323	0.0762	0.7863						
	0.9154	0.6774	0.1063						
	0.9217	0.8603	-0.0983						
IPC_sets	0.0509	0.0173	1.9601	$F_{(9,305)} = 1.84$	421.29	0	0.0996	0.0608	intrcpt
	0.0144	0.4863	2.4619						0.049
	0.971	NA	-0.0364						
	0.3703	0.0685	0.8972						
	0.847	0.0018	-0.1931						
	0.0149	0.003	2.4491						
	0.7264	0.3094	0.3502						
	0.585	NA	0.5466						
	0.9361	0.6456	0.0802						
Warm-up	0.1716	0.2467	1.3703	$F_{(2,312)} = 6.156$	426.052	0	0.0945	0.0542	0.5884
	**0.0011**	**0.0004**	3.2928						

K: number of studies; n:Total number of effects included; 95%CI: Unadjusted 95% confidence interval (range of effect size estimates); 95%CI_adj: Adjusted 95% confidence interval (corrected for multilevel models or heterogeneity); p-value_adj: Adjusted p-value (e.g., corrected for multiple comparisons or model adjustments); t-value: t-test statistic (for testing significance of the effect size); Q test: Q statistic for heterogeneity test (assessing between-study heterogeneity); I^2-Level 2/3: Heterogeneity proportions in multilevel models; PI: Prediction Interval; *p*-Egger: Egger test p-value to detect publication bias (e.g. $p < 0.05$ for possible bias);Moderator: Subgroup category (e.g., intervention type, population characteristics); F-test: F-statistic for ANOVA or model significance; $p_{\text{-diff}}$: p-value for subgroup differences; intrcpt: Intercept term in the model (baseline effect); NA: Data not available or not applicable; CON: Control group (no intervention); SHAM: Sham intervention group (sham or simulated intervention).

TABLE S14. Subgroup analyses of IPC on anaerobic sport performance—Three or Four-level model (1)

Variable	Moderator	Studies(K)	Effect size(n)	Effect size	95%CI	95%CI_adj
**Comparison**	IPC *vs.* CON	15	42	0.2165	[0.0659; 0.3672]	[0.0378; 0.3953]
	IPC *vs.* SHAM	45	131	0.1256	[0.0229; 0.2283]	[0.0390; 0.2122]
**Sex**	female	4	10	0.1085	[-0.1542; 0.3711]	[-0.2113; 0.4283]
	male	31	100	0.2212	[0.0932; 0.3493]	[0.1134; 0.3291]
	mixed	16	58	0.0877	[-0.0614; 0.2367]	[0.0071; 0.1682]
	NA	2	5	-0.261	[-0.7449; 0.2229]	[-6.7358; 6.2137]
**Experence_level**	level0	6	16	0.1568	[-0.1456; 0.4592]	[-0.0661; 0.3798]
	level1	16	72	0.1557	[-0.0044; 0.3158]	[0.0188; 0.2926]
	level2	15	51	0.2151	[0.0343; 0.3959]	[-0.0103; 0.4406]
	level3	4	14	0.1435	[-0.2234; 0.5104]	[-0.0827; 0.3697]
	level4	5	15	-0.0264	[-0.3623; 0.3095]	[-0.0519; -0.0010]
	level5	3	5	-0.0592	[-0.4860; 0.3677]	[-0.4253; 0.3070]
**IPC_sets**	1 × 5 min	1	1	1.6166	[0.6521; 2.5810]	[NA; NA]
	3 × 5 min	26	85	0.1279	[-0.0121; 0.2679]	[0.0438; 0.2119]
	4 × 5 min	21	86	0.1293	[-0.0133; 0.2720]	[-0.0162; 0.2749]
	5 × 5 min	1	1	0.1584	[-0.3853; 0.7020]	[NA; NA]
**Warm-up**	NO	9	35	0.0815	[-0.1290; 0.2920]	[-0.0569; 0.2199]
	YES	40	138	0.1629	[0.0517; 0.2740]	[0.0638; 0.2619]
**Outcome**	Balance	1	1	0.1584	[-0.3825; 0.6992]	[NA; NA]
	Jump	4	8	0.0526	[-0.2318; 0.3369]	[-0.4893; 0.5944]
	MAOD	1	1	-0.1275	[-0.6833; 0.4282]	[-0.4533; 0.1983]
	P	13	46	0.045	[-0.0873; 0.1774]	[-0.0542; 0.1443]
	RM	12	30	0.4262	[0.2188; 0.6337]	[0.1157; 0.7368]
	Strength	14	40	0.0617	[-0.0792; 0.2027]	[-0.0631; 0.1866]
	Time to complete	15	32	0.1148	[-0.0404; 0.2701]	[-0.0078; 0.2375]
	Time to failure	3	10	0.4555	[0.0073; 0.9036]	[-1.4668; 2.3777]
	$\dot{V}O_2$	3	5	-0.1383	[-0.4383; 0.1617]	[-0.9128; 0.6362]

TABLE S14. Subgroup analyses of IPC on anaerobic sport performance—Three or Four-level model (2)

Variable	p-value	p-value_adj	t-value	F-test	Q test	I^2-Level 2	I^2-Level 3	$p_{\text{-diff}}$
Comparison	**0.0051**	**0.021**	2.8373	F (2 , 171) = 5.029	240.901	0	0.1326	0.2187
	**0.0168**	**0.0056**	2.4143					
Sex	0.4161	0.3615	0.8152	F (4 , 169) = 3.434	236.077	0	0.1246	no difference
	**0.0008**	**0.0002**	3.4119					
	0.2471	0.0356	1.1614					
	0.2884	0.6989	-1.0649					
Experence_level	0.3074	0.1248	1.0239	F (6 , 167) = 1.825	239.265	0	0.1471	no difference
	0.0566	0.0293	1.9198					
	0.02	0.0598	2.3493					
	0.4411	0.1336	0.7722					
	0.8767	0.0451	-0.1554					
	0.7847	0.5283	-0.2736					
IPC_sets	0.0011	NA	3.3089	F (4 , 169) = 4.433	232.091	0	0.1297	**1 × 5**
	0.0731	0.0046	1.8031					0.003
	0.0753	0.0778	1.7895					0.003
	0.566	NA	0.575					0.0101
Warm-up	0.4458	0.1909	0.7642	F (2 , 171) = 4.475	241.445	0	0.138	
	**0.0043**	**0.002**	2.8925					0.5007
Outcome	0.564	NA	0.5782	F (9 , 164) = 2.722	225.127	0	0.1244	
	0.7156	0.7711	0.365					
	0.6511	0.2524	-0.453					
	0.5026	0.3434	0.6719					
	**0.0001**	**0.012**	4.0565					
	0.3883	0.2958	0.8649					
	0.1461	0.0644	1.4603					
	0.0464	0.4032	2.0068					
	0.364	0.5566	-0.9103					

K: number of studies; n:Total number of effects included 95%CI: Unadjusted 95% confidence interval (range of effect size estimates); 95%CI_adj: Adjusted 95% confidence interval (corrected for multilevel models or heterogeneity); p-value_adj: Adjusted p-value (e.g., corrected for multiple comparisons or model adjustments); t-value: t-test statistic (for testing significance of the effect size); Q test: Q statistic for heterogeneity test (assessing between-study heterogeneity); I^2-Level 2/3: Heterogeneity proportions in multilevel models; PI: Prediction Interval; Moderator: Subgroup category (e.g., intervention type, population characteristics); F-test: F-statistic for ANOVA or model significance; p-diff: p-value for subgroup differences; intrcpt: Intercept term in the model (baseline effect); NA: Data not available or not applicable; CON: Control group (no intervention); SHAM: Sham intervention group (sham or simulated intervention). MAOD: Maximum acumulative oxygen deficit; P: power output; RM: Maximum number of repetitions of the Exhaustion Test; $\dot{V}O_2$: oxygen uptake;

TABLE S15. Effects of IPC on aerobic sport performance—Three or Four-level model (1)

Variable	Moderator	Studies(K)	Effect size(n)	Effect size	95%CI	95%CI_adj
Comparison	IPC *vs.* CON	17	53	0.21	[0.0479; 0.3720]	[-0.0041; 0.4240]
	IPC *vs.* SHAM	31	88	0.0413	[-0.0858; 0.1684]	[-0.0318; 0.1145]
Sex	female	3	7	0.0989	[-0.2159; 0.4137]	[-0.3539; 0.5517]
	male	23	82	0.1483	[-0.0005; 0.2971]	[0.0098; 0.2868]
	mixed	15	39	0.0309	[-0.1643; 0.2260]	[-0.1062; 0.1679]
	NA	3	13	0.0391	[-0.4331; 0.5112]	[-0.2763; 0.3544]
Experence_level	level0	4	19	0.1089	[-0.2892; 0.5070]	[-0.0204; 0.2382]
	level1	13	44	0.1098	[-0.0821; 0.3016]	[-0.1577; 0.3772]
	level2	9	25	0.1322	[-0.1014; 0.3658]	[0.0389; 0.2255]
	level3	11	29	0.0574	[-0.1880; 0.3028]	[-0.0601; 0.1749]
	level4	5	22	0.0537	[-0.2942; 0.4017]	[-0.1403; 0.2478]
	level5	1	2	0.1552	[-0.4609; 0.7712]	[0.1445; 0.1659]
IPC_sets	1 × 5 min	1	6	0.2728	[-0.2436; 0.7892]	[-0.2386; 0.7842]
	3 × 10 min	1	3	0.143	[-0.3155; 0.6015]	[-0.2298; 0.5158]
	3 × 2 min	1	1	-0.0166	[-0.9374; 0.9042]	[NA; NA]
	3 × 3 min + 2 min reperfusion	1	2	-0.0613	[-0.6790; 0.5563]	[-0.0623; -0.0604]
	3 × 5 min	15	53	0.1298	[-0.0610; 0.3205]	[-0.0997; 0.3592]
	4 × 5 min	24	71	0.077	[-0.0782; 0.2322]	[-0.0120; 0.1661]
	5 × 2 min	1	3	0.1364	[-0.5965; 0.8693]	[-0.3581; 0.6309]
	8 × 5 min	1	2	-0.0084	[-0.5499; 0.5332]	[-0.1227; 0.1060]
Warm-up	NO	12	49	0.0907	[-0.1095; 0.2908]	[-0.2248; 0.4061]
	YES	33	92	0.1028	[-0.0265; 0.2320]	[-0.0013; 0.2069]
Outcome	Time to failure	8	11	0.5147	[0.2738; 0.7556]	[0.1570; 0.8724]
	P	16	30	0.098	[-0.0593; 0.2554]	[0.0035; 0.1925]
	Strength	4	19	0.026	[-0.2888; 0.3408]	[-0.2595; 0.3114]
	Time to complete	20	35	0.0728	[-0.0811; 0.2268]	[-0.1460; 0.2916]
	$\dot{V}O_2$	22	46	0.0131	[-0.1303; 0.1564]	[-0.1345; 0.1606]

K: number of studies; n:Total number of effects included; 95%CI: Unadjusted 95% confidence interval (range of effect size estimates); 95%CI_adj: Adjusted 95% confidence interval (corrected for multilevel models or heterogeneity); p-value_adj: Adjusted p-value (e.g., corrected for multiple comparisons or model adjustments); t-value: t-test statistic (for testing significance of the effect size); Q test: Q statistic for heterogeneity test (assessing between-study heterogeneity); I^2-Level 2/3/4: Heterogeneity proportions in multilevel models; PI: Prediction Interval; Moderator: Subgroup category (e.g., intervention type, population characteristics); F-test: F-statistic for ANOVA or model significance; $p_{\text{-diff}}$: p-value for subgroup differences; intrcpt: Intercept term in the model (baseline effect); NA: Data not available or not applicable; CON: Control group (no intervention); SHAM: Sham intervention group (sham or simulated intervention); P: power output; $\dot{V}O_2$: oxygen uptake;

TABLE S15. Effects of IPC on aerobic sport performance—Three or Four-level model (2)

Variable	p-value	p-value_adj	t-value	F-test	Q test	I^2-Level 2	I^2-Level 3	$p_{\text{-diff}}$
**Comparison**	0.0115	0.054	2.5622	F (2 , 139) = 3.283	175.234	0	0.153	0.0638
	0.5214	0.2573	0.6428					
**Sex**	0.5354	0.4324	0.6213	F (4 , 137) = 1.016	179.682	0	0.1698	no difference
	0.0507	0.037	1.9713					
	0.755	0.6328	0.3126					
	0.8702	0.6423	0.1637					
**Experence_level**	0.5895	0.0747	0.5409	F (6 , 135) = 0.564	180.55	0	0.171	no difference
	0.2598	0.3889	1.1316					
	0.2649	0.0121	1.1194					
	0.6444	0.2965	0.4626					
	0.7604	0.4654	0.3055					
	0.6192	0.0034	0.4982					
**IPC_sets**	0.298	0.0933	1.0448	F (8 , 133) = 0.52	179.552	0	0.1771	no difference
	0.5384	0.2775	0.6168					
	0.9716	NA	-0.0356					
	0.8446	0.0008	-0.1964					
	0.1806	0.244	1.3458					
	0.328	0.0864	0.9817					
	0.7133	0.1769	0.3682					
	0.9756	0.8257	-0.0306					
**Warm-up**	0.3719	0.5391	0.8958	F (2 , 139) = 1.543	180.347	0	0.1658	no difference
	0.1182	0.0528	1.572					
**Outcome**	**0**	**0.0106**	4.2256	F (5 , 136) = 4.023	158.512	0	0.1275	
	0.2202	0.0428	1.2317					
	0.8706	0.7943	0.1632					
	0.3512	0.4961	0.9355					
	0.8573	0.8569	0.1801					

K: number of studies; n:Total number of effects included; 95%CI: Unadjusted 95% confidence interval (range of effect size estimates); 95%CI_adj: Adjusted 95% confidence interval (corrected for multilevel models or heterogeneity); p-value_adj: Adjusted p-value (e.g., corrected for multiple comparisons or model adjustments); t-value: t-test statistic (for testing significance of the effect size); Q test: Q statistic for heterogeneity test (assessing between-study heterogeneity); I^2-Level 2/3/4: Heterogeneity proportions in multilevel models; PI: Prediction Interval; Moderator: Subgroup category (e.g., intervention type, population characteristics); F-test: F-statistic for ANOVA or model significance; $p_{\text{-diff}}$: p-value for subgroup differences; intrcpt: Intercept term in the model (baseline effect); NA: Data not available or not applicable; CON: Control group (no intervention); SHAM: Sham intervention group (sham or simulated intervention); P: power output; $\dot{V}O_2$: oxygen uptake;

Model Results:

	estimate	se	tval	df	pval	ci.lb	ci.ub	
intrcpt	0.4262	0.1051	4.0565	164	<.0001	0.2188	0.6337	***
OutcomeBalance	-0.2679	0.2934	-0.9131	164	0.3625	-0.8471	0.3114	
OutcomeJump	-0.3737	0.1760	-2.1226	164	0.0353	-0.7212	-0.0261	*
OutcomeMAOD	-0.5537	0.2990	-1.8516	164	0.0659	-1.1442	0.0367	.
OutcomeP	-0.3812	0.1190	-3.2022	164	0.0016	-0.6162	-0.1461	**
OutcomeStrength	-0.3645	0.1173	-3.1068	164	0.0022	-0.5961	-0.1328	**
OutcomeTime to complete	-0.3114	0.1289	-2.4152	164	0.0168	-0.5659	-0.0568	*
OutcomeTime to failure	0.0293	0.2488	0.1176	164	0.9065	-0.4620	0.5205	
OutcomeV̇O2	-0.5645	0.1827	-3.0906	164	0.0023	-0.9252	-0.2039	**

FIG. S4. Subgroup analysis of anaerobic performance with “RM” as the intercept.

Model Results:

	estimate	se	tval	df	pval	ci.lb	ci.ub	
intrcpt	0.5147	0.1218	4.2256	136	<.0001	0.2738	0.7556	***
OutcomeP	-0.4167	0.1327	-3.1402	136	0.0021	-0.6791	-0.1543	**
OutcomeStrength	-0.4887	0.1803	-2.7105	136	0.0076	-0.8453	-0.1321	**
OutcomeTime to complete	-0.4419	0.1359	-3.2523	136	0.0014	-0.7105	-0.1732	**
OutcomeV̇O2	-0.5016	0.1262	-3.9741	136	0.0001	-0.7512	-0.2520	***

FIG. S5. Subgroup analysis of aerobic performance with “Time to failure” as the intercept.

ELECTRONIC SUPPLEMENTARY MATERIAL APPENDIX S8 (FUNNEL PLOTS WITH EGGER'S TEST FOR EXERCISE OUTCOMES ACROSS DIFFERENT ENERGY SYSTEMS)

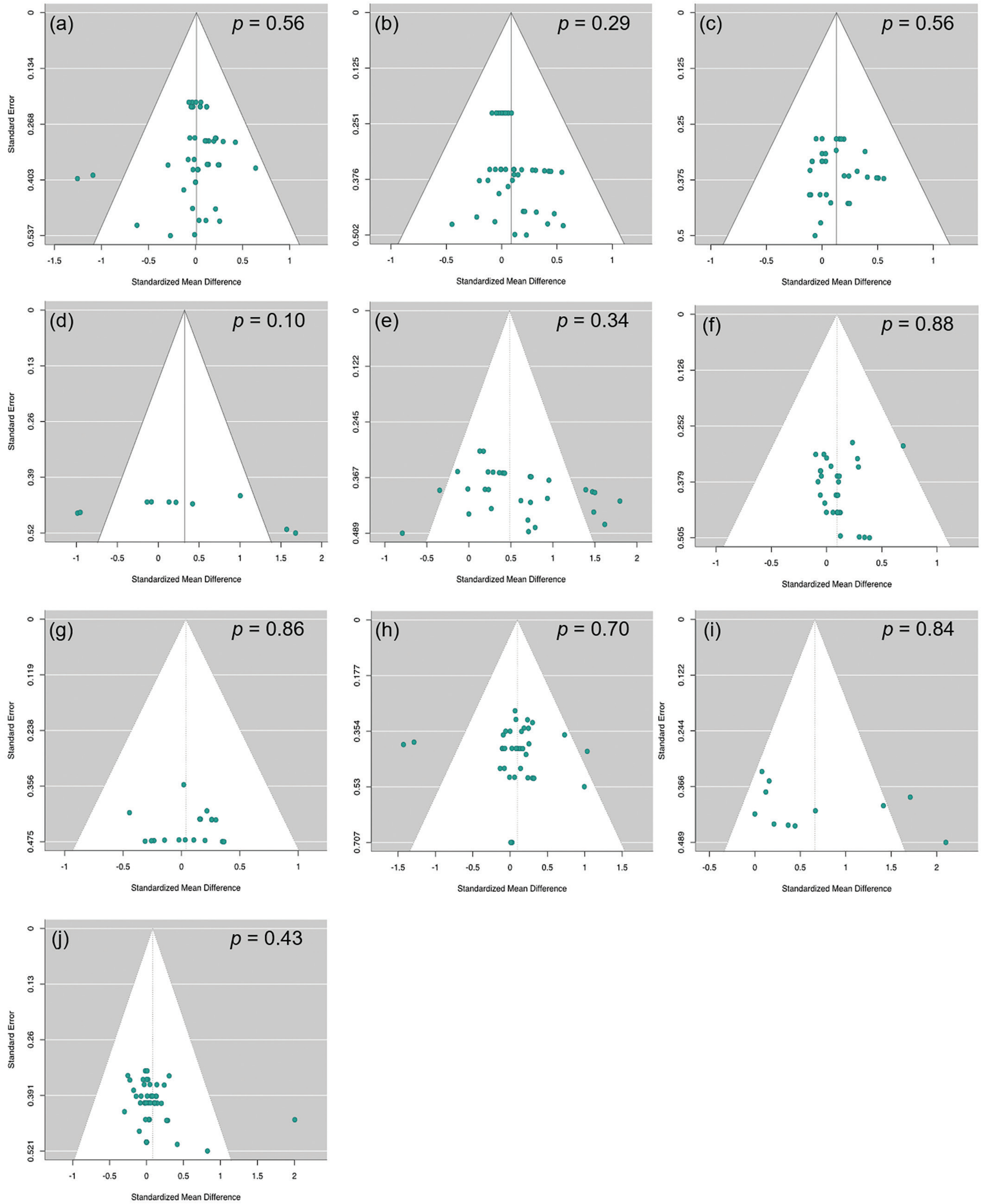

FIG. S6. Funnel plots with Egger's test for exercise outcomes across different energy systems. (a)–(e): Funnel plots for anaerobic performance, representing power output, strength, time to complete, time to failure, and maximal number of repetitions, respectively; (f)–(j): Funnel plots for aerobic performance, representing power output, strength, time to complete, time to failure, and $\dot{V}O_2$, respectively. *P*-values correspond to the results of Egger's test, with values < 0.05 indicating the presence of publication bias.

Articles published in the Biology of Sport are licensed under an open access Creative Commons CC BY 4.0 license.
